# The RAC1 activator Tiam1 regulates centriole duplication through controlling PLK4 levels

**DOI:** 10.1242/jcs.252502

**Published:** 2021-04-15

**Authors:** Andrew P. Porter, Hannah Reed, Gavin R. M. White, Erinn-Lee Ogg, Helen J. Whalley, Angeliki Malliri

**Affiliations:** Cell Signalling Group, Cancer Research UK Manchester Institute, The University of Manchester, Alderley Park, Macclesfield SK10 4TG, UK

**Keywords:** Centriole, Centriole duplication, PLK4, Tiam1, βTRCP, Aneuploidy

## Abstract

Centriole duplication is tightly controlled to maintain correct centriole number through the cell cycle. Key to this is the regulated degradation of PLK4, the master regulator of centriole duplication. Here, we show that the Rac1 guanine nucleotide exchange factor (GEF) Tiam1 localises to centrosomes during S-phase, where it is required for the maintenance of normal centriole number. Depletion of Tiam1 leads to an increase in centrosomal PLK4 and centriole overduplication, whereas overexpression of Tiam1 can restrict centriole overduplication. Ultimately, Tiam1 depletion leads to lagging chromosomes at anaphase and aneuploidy, which are potential drivers of malignant progression. The effects of Tiam1 depletion on centrosomal PLK4 levels and centriole overduplication can be rescued by re-expression of both wild-type Tiam1 and catalytically inactive (GEF*) Tiam1, but not by Tiam1 mutants unable to bind to the F-box protein βTRCP (also known as F-box/WD repeat-containing protein 1A) implying that Tiam1 regulates PLK4 levels through promoting βTRCP-mediated degradation independently of Rac1 activation.

## Abstract (precis)

**Summary:** Tiam1 maintains centriole number and ensures correct chromosome segregation via interaction with βTRCP, part of the E3 ligase complex, which degrades PLK4, the master regulator of centriole duplication.

## INTRODUCTION

Centrioles are barrel-shaped microtubule-rich organelles fundamental to cell division, polarity and signalling. Centriole pairs recruit pericentriolar material (PCM) to form the centrosome, the major microtubule organising centre (Conduit et al., 2015). In G1, a typical human cell contains a single centrosome formed around two centrioles. At the G1/S transition, each of the mother centrioles generates a single daughter centriole (see schematic, Fig. S1A), which together recruit PCM to form one of the mitotic spindle poles (Banterle and Gönczy, 2017).

Centriole number is tightly controlled. Increased centriole or centrosome number leads to severe mitotic aberrations (Nigg et al., 2014; Banterle and Gönczy, 2017). During mitosis, cells with centrosome amplification can form transient multipolar spindles. Divisions proceed in such cells through clustering of extra centrosomes to form pseudo-bipolar spindles. However, the formation of multipolar intermediates increases the incidence of merotelic microtubule-kinetochore attachments to chromosomes that do not trigger the spindle assembly checkpoint. These in turn can give rise to lagging chromosomes and ultimately aneuploidy (Ganem et al., 2009). Moreover, even in cells with two centrosomes, uneven centriole number at the mitotic poles can lead to lagging chromosomes and aneuploidy (Cosenza et al., 2017). In turn, aneuploidy leads to transcriptional changes that can affect cell growth or cause proteotoxic stress. Aneuploidy is also a feature of chromosomal instability (CIN), a driver of cancer (Nigg et al., 2014). Indeed, it has been reported that centrosome amplification alone is sufficient for the initiation of tumourigenesis (Levine et al., 2017).

To ensure correct centriole number, cells tightly regulate centriole production (Banterle and Gönczy, 2017; Nigg and Holland, 2018). Central to this is PLK4, a member of the polo-like kinase family, the master regulator of centriole biogenesis (Nigg and Holland, 2018). During the G1-S transition, PLK4 phosphorylates its partner STIL, increasing recruitment of SAS6 (also known as SASS6), a key structural component of the new procentriole (Fig. S1A) (Nigg and Holland, 2018). This process is restricted by PLK4 degradation, which is mediated by the SCF E3 ligase complex. PLK4 proteins homodimerise and trans-autophosphorylate a phospho-degron recognised by βTRCP (also known as F-box/WD repeat-containing protein 1A), an F-box protein that targets SCF to its substrates (Guderian et al., 2010; Holland et al., 2010, 2012b). Thus, a transient increase in PLK4 activity is tightly coupled to its destruction, efficiently terminating its activity and preventing extra rounds of centriole duplication (Holland et al., 2010, 2012b). Excess PLK4 has been shown to lead to centriole overduplication across multiple species and cell types (Guderian et al., 2010; Holland et al., 2010; Arquint and Nigg, 2016; Basto et al., 2008; Kleylein-Sohn et al., 2007).

Tiam1, a guanine nucleotide exchange factor (GEF) for the small GTPase Rac1, has a wide variety of functions, including roles in cell adhesion, polarity, migration, transcription and tumourigenesis (Malliri et al., 2002, 2004, 2006; Mack et al., 2012; Vaughan et al., 2014; Diamantopoulou et al., 2017; Maltas et al., 2020). We previously showed that Tiam1 localises to centrosomes in prophase and prometaphase, where it activates Rac1 signalling to antagonise centrosome separation (Woodcock et al., 2010). Centrosomal Tiam1 is phosphorylated by CDK1 and this is required for phosphorylation and consequently activation of the Rac1 effector PAK1/2 (Whalley et al., 2015). In this study we demonstrate a new centrosomal role for Tiam1 earlier in the cell cycle, in the control of centriole duplication. We show that Tiam1 is required for the maintenance of normal PLK4 protein levels, and that this involves an interaction with βTRCP but not the activation of Rac1.

## RESULTS

**Fig. 1.**
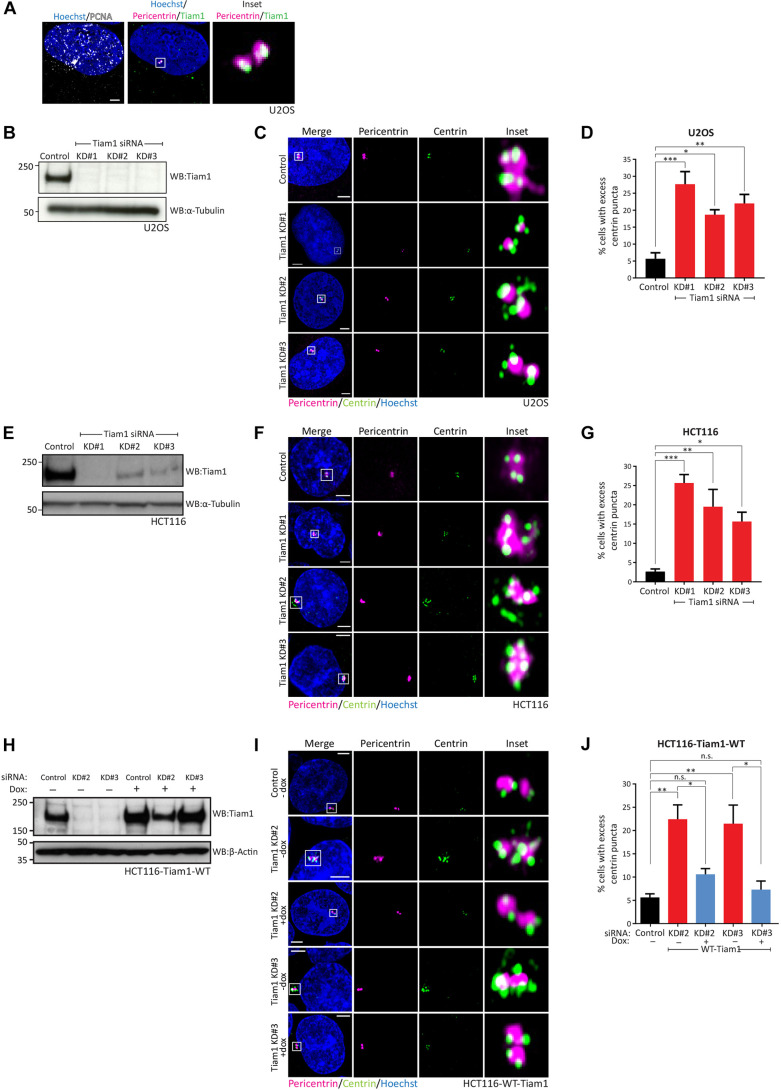
**Depletion of Tiam1 leads to the formation of excess centrin puncta.** (A) Endogenous Tiam1 (green) localises to centrosomes (marked by pericentrin, magenta) during S-phase (identified by punctate nuclear staining of PCNA, grey). Maximal *z*-projection of AiryScan confocal image planes containing pericentrin staining. Panel labelled ‘Inset’ shows a magnified version of the region marked with a white box in the merge, with Hoechst omitted for clarity. (B) WB showing depletion of endogenous Tiam1 in U2OS cells following transfection with three independent siRNAs. (C) Maximal *z*-projections of AiryScan confocal images showing normal centrin puncta (green, maximum of two per centrosome) in U2OS cells transfected with control siRNA, and excess centrin puncta in Tiam1-knockdown cells. Centrosomes are marked with pericentrin (magenta). Panels labelled ‘Inset’ show a magnified version of the region marked with a white box in the merge, with Hoechst omitted for clarity; the same principle applies to F and I. (D) Quantification of U2OS cells with excess centrin puncta from three independent experiments, quantified from images as in C. More than 100 cells counted per condition per experiment. (E) WB showing depletion of endogenous Tiam1 in HCT116 cells following transfection with three independent siRNAs. In B and E, α-tubulin was used as a loading control. (F) Representative AiryScan confocal microscopy images showing normal centrin puncta (green) in HCT116 cells transfected with control siRNA, and excess centrin puncta in Tiam1 knockdown cells. Centrosomes are marked with pericentrin (magenta). (G) Quantification of HCT116 cells with excess centrin puncta from three independent experiments (except Tiam1 KD#2, *n*=2), quantified from images as in F. More than 100 cells were counted per condition per experiment. (H) WB from HCT116-Tiam1-WT cells showing depletion of endogenous Tiam1 following siRNA transfection with Tiam1 KD#2 and KD#3, and restoration of near-endogenous levels of Tiam1 following treatment with doxycycline (dox) to induce expression of siRNA-resistant wild-type (WT) Tiam1. β-actin was used as a loading control. (I) Representative AiryScan confocal images of control, Tiam1 knockdown and rescue cells showing excess centrin puncta following Tiam1 knockdown and restoration of normal centrin puncta (maximum of two per centrosome) in cells re-expressing wild-type Tiam1. (J) Quantification of images as in I from four independent experiments. More than 50 cells were counted per condition per experiment. Data are mean±s.e.m. Statistical significance was determined using one-way ANOVA, corrected for multiple comparisons. A two-tailed, unpaired *t*-test was used for comparing +/− dox cells treated with the same siRNA. *P<0.05; **P<0.01; ***P<0.001; n.s., not significant. Scale bars: 3 µm.

We decided to investigate whether Tiam1 localises to centrosomes in other phases of the cell cycle besides mitosis. We used our previously validated staining protocol (Woodcock et al., 2010) and stained U2OS cells with antibodies against Tiam1, pericentrin (as a centrosome marker) and PCNA, which produces punctate nuclear staining during S-phase (Schönenberger et al., 2015). We observed localisation of Tiam1 at centrosomes during S-phase (Fig. 1A), as well as throughout mitosis as we have described previously (Woodcock et al., 2010) (data not shown).

We hypothesized that Tiam1 plays an additional role in centrosome biology in S-phase, when centriole duplication occurs (Nigg and Holland, 2018). To test this, we depleted Tiam1 from U2OS cells using RNAi (Fig. 1B). An asynchronous population of control and knockdown cells were stained with antibodies against pericentrin, to mark the pericentriolar material surrounding the centrioles (Doxsey et al., 1994), and centrin (herein referring generically to all isoforms), an integral structural component of centrioles themselves (Bornens, 2002; Fig. 1C). Depletion of Tiam1 led to an increase in the percentage of cells displaying excess centrin puncta (i.e. greater than two centrin puncta per centrosome) (Fig. 1C). Using three independent siRNA sequences targeting Tiam1, we saw a significant increase in the number of cells displaying these additional centrin puncta compared with control-treated cells (Fig. 1D). To determine whether this effect could be observed in other cell lines, we used siRNA to deplete Tiam1 in HCT116 colon cancer cells, which, like U2OS cells, have normal centriole numbers (Cosenza et al., 2017; Marteil et al., 2018). Tiam1 depletion (Fig. 1E) again resulted in an increase in cells displaying excess centrin puncta compared with control siRNA (Fig. 1F,G). This suggests a conserved role for Tiam1 in maintaining normal centrosome structure across cell types with very different tissues of origin.

To demonstrate that the effects of Tiam1 depletion are on-target effects of siRNA knockdown, we developed a system to restore wild-type Tiam1 levels using siRNA-resistant Tiam1. We constructed HCT116 cells expressing mouse wild-type Tiam1 [WT-Tiam1; intrinsically resistant to two siRNAs targeting the human Tiam1 sequence (Tiam1 KD#2 and Tiam2 KD#3)] under the control of a doxycycline-inducible promoter, as described previously (Mack et al., 2012; Whalley et al., 2015). We could detect the exogenous WT-Tiam1 localising to centrosomes by immunofluorescence (IF) following doxycycline treatment (Fig. S1B). As before, siRNA treatment effectively depleted endogenous Tiam1; however, the addition of doxycycline led to near-endogenous expression of WT-Tiam1 protein (Fig. 1H). Although Tiam1 depletion again led to an increase in cells containing centrosomes with excess centrin puncta, this was effectively rescued by doxycycline-induced WT-Tiam1 (representative images in Fig. 1I, quantified in Fig. 1J), demonstrating the specificity of the knockdown and that this phenotype is due to the loss of Tiam1 itself.

**Fig. 2.**
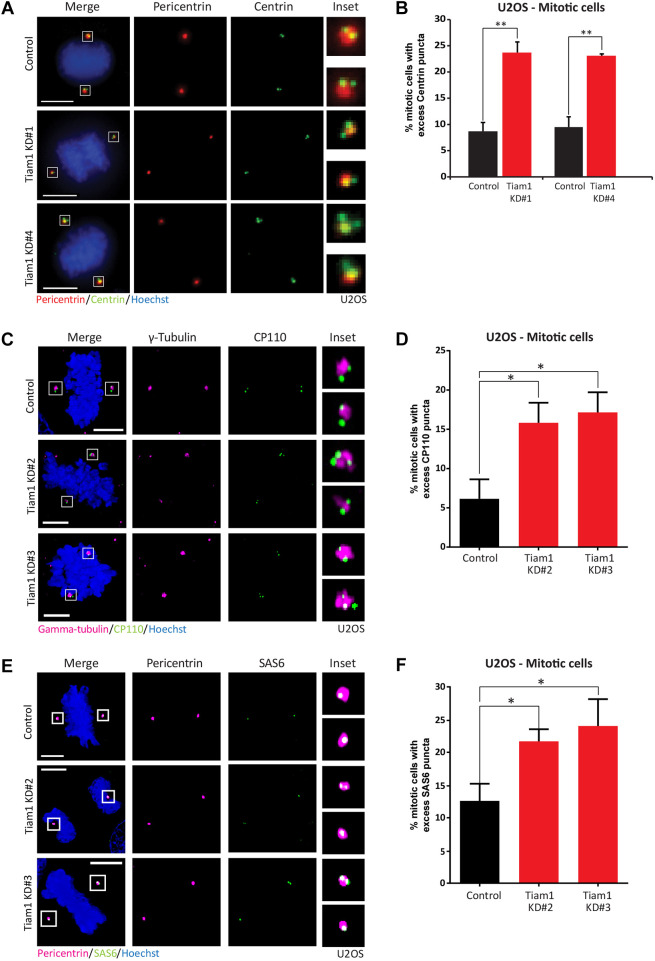
**Depletion of Tiam1 induces excess SAS6 and CP110 puncta.** (A) Deltavision maximal *z*-projection images of mitotic U2OS cells treated with either control or Tiam1 KD#1 or KD#4 siRNA, showing an increase in excess centrin puncta (green) in Tiam1 knockdown cells. Centrosomes are marked by pericentrin (red). (B) Quantification of cells with excess centrin puncta, quantified from images in A, from three independent experiments (Tiam1 KD#1: 100 mitotic cells quantified per experimental replicate; Tiam1 KD#4: ≥45 mitotic cells quantified per experimental replicate). (C) AiryScan confocal images of mitotic U2OS cells stained with CP110 (green), showing excess CP110 puncta (greater than two puncta per centrosome) following Tiam1 knockdown. Centrosomes are marked by γ-tubulin (magenta). (D) Quantification of mitotic U2OS cells with excess CP110 puncta from four independent experiments, quantified from images in C. An average of 45 cells were counted per condition per experiment. (E) AiryScan confocal images of mitotic U2OS cells stained with SAS6 (green), showing excess SAS6 puncta (greater than one puncta per centrosome) following Tiam1 knockdown. Centrosomes are marked by pericentrin (magenta). (F) Quantification of mitotic U2OS cells with excess SAS6 puncta from four independent experiments, quantified from images in E. An average of 38 cells were counted per condition per experiment. Data are mean±s.e.m. *P<0.05; **P<0.01 (two-tailed, paired *t*-test). Scale bars: 10 µm (A); 5 µm (C,E).

Next, we sought to determine whether the increase in cells with extra centrin puncta marked a genuine increase in centrioles. First, we tested whether mitotic cells contained centrosomes with excess centrin puncta, which would indicate that these abnormal centrioles were incorporated into centrosomes capable of nucleating microtubules and forming the poles of the mitotic spindle. We knocked down Tiam1 in U2OS cells (Fig. S2A) and specifically assessed centrin puncta in mitotic cells by widefield imaging. Any cell with a centrosome (marked with pericentrin) containing more than two centrin puncta was classed as abnormal (Fig. 2A). Around a quarter of all Tiam1-depleted mitotic U2OS cells had at least one abnormal centrosome at the pole of the mitotic spindle, a significant increase for both siRNAs compared with control cells (Fig. 2A, quantified in Fig. 2B).

We then looked at whether other commonly used markers of centrioles showed changes following Tiam1 knockdown. We stained U2OS cells with CP110, which caps the ends of developed centrioles (Kleylein-Sohn et al., 2007; Chen et al., 2002) (Fig. S1A). We again looked specifically at mitotic cells, as this provides a matched population that have been through at least one round of S-phase, the period of the cell cycle in which centriole duplication occurs. AiryScan confocal imaging of control cells showed two puncta of CP110 at each pole of the mitotic spindle [marked by γ-tubulin staining (Moritz et al., 2000); Fig. 2C]. Depletion of Tiam1 with two independent siRNAs led to a significant increase in mitotic cells with excess CP110 puncta compared with control-treated cells (Fig. 2D). The increase in the percentage of abnormal cells was comparable to the increase in cells quantified using centrin staining (Fig. 2A,B).

We used a third distinct centriole marker to assess centriolar abnormalities following Tiam1 depletion. We chose SAS6, a key structural component of the cartwheel that forms the base of the developing pro-centriole in S-phase (Fig. S1) (Arquint and Nigg, 2016; Leidel et al., 2005). Using a mitotic population of U2OS cells, we saw through AiryScan confocal imaging that mitotic centrosomes in control cells (marked by pericentrin staining) typically contained a single puncta of SAS6 (Fig. 2E). Depletion of Tiam1 led to a significant increase in cells with centrosomes containing excess puncta of SAS6 (Fig. 2F).

As an additional test for whether these excess puncta marked true centrioles, we performed an assay to disrupt centriolar satellites, which localise around the centrosome and also stain with centrin antibody (Bärenz et al., 2011). We placed control and Tiam1 knockdown cells on ice for 30 min to depolymerise microtubules. Although this causes dispersion of centriolar satellites, true centrioles are unaffected (Kubo et al., 1999; Tsang et al., 2009). We continued to observe a significant increase in Tiam1 knockdown cells with excess centrin puncta following cold treatment (Fig. S2B,C), indicating that the extra centrin puncta are indeed incorporated into stable centrioles.

Furthermore, we reviewed our images to look for evidence of centrosome amplification, which we would expect to occur as a consequence of centriole overduplication. For both U2OS and HCT116 cell lines, we observed a small increase in the number of cells displaying centrosome amplification following Tiam1 knockdown (representative images in Fig. S2D and quantification in Fig. S2E).

Given the increase in active mitotic centrosomes containing excess puncta of three centriole markers marking distinct components of the centriole, and that the centrin puncta are stable after cold treatment, and that we see a small increase in centrosome amplification, we propose that Tiam1 depletion leads to centriole overduplication.

We next tested whether the overexpression of Tiam1 is capable of restraining excess centriole duplication. Treatment with hydroxyurea results in a prolonged S-phase, leading to centriole overduplication (Balczon et al., 1995; Lončarek et al., 2010), which we can detect through an increase in centrin staining. Expression of exogenous, GFP-tagged WT-Tiam1 (Fig. S2F) was able to significantly reduce the number of hydroxyurea-treated U2OS cells displaying excess centrin puncta compared with a GFP-only control (Fig. S2G,H). This suggests that Tiam1 plays a role in limiting centriole duplication.

To determine the mechanism by which Tiam1 maintains normal centriole number, we first used flow cytometry to investigate whether Tiam1 knockdown affected cell cycle progression, as increased S-phase duration can lead to centriole overduplication (Balczon et al., 1995), and failure to undergo cytokinesis results in centrosome amplification (Cosenza and Krämer, 2016). However, we saw no difference in cell cycle progression or evidence of polyploidy comparing control and Tiam1 knockdown cells (Fig. S3A-C). Having excluded a cell cycle defect, we decided to next examine whether Tiam1 knockdown affected PLK4 levels at centrosomes, with PLK4 being the key driver of centriole duplication (Guderian et al., 2010; Holland et al., 2010; Arquint and Nigg, 2016; Basto et al., 2008; Kleylein-Sohn et al., 2007). We first confirmed that our antibody detected centrosomal PLK4 by widefield imaging of U2OS cells treated with either a control siRNA or several PLK4 siRNAs (Fig. S3D). We calculated PLK4 centrosomal intensity (Fig. S3E) and saw a decrease in centrosomal PLK4 in all siRNA-treated cells compared with control cells (Fig. S3F).

**Fig. 3.**
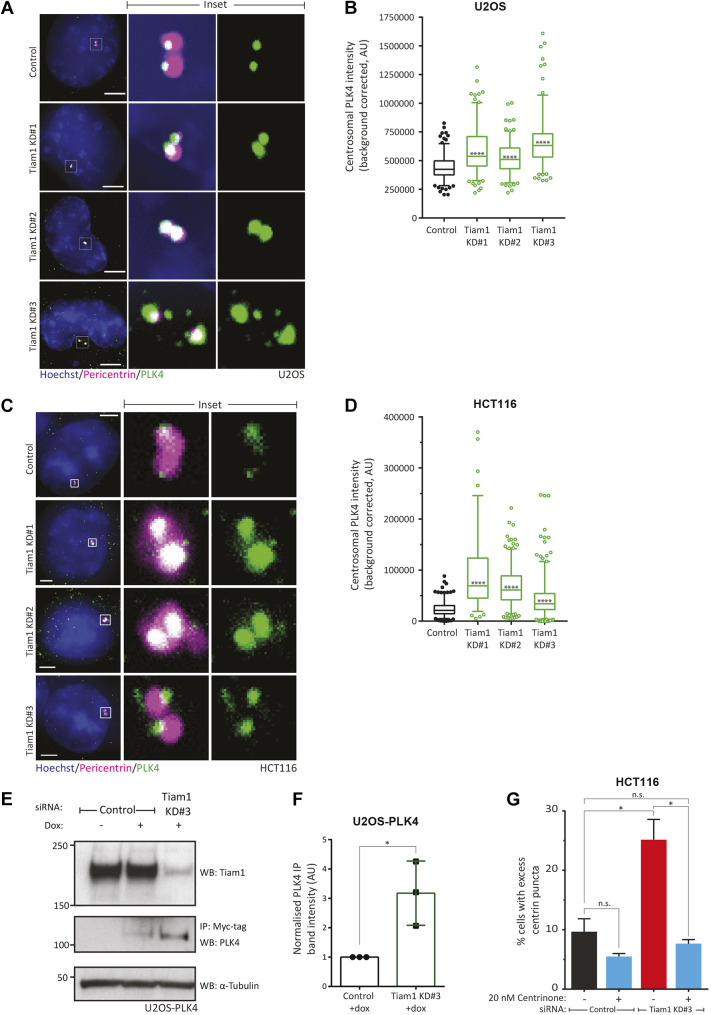
**Tiam1 knockdown leads to an increase in centrosomal PLK4.** (A) Maximal *z*-projections of U2OS cells stained for pericentrin (magenta) as a marker of centrosomes, and endogenous PLK4 (green), showing an increase in centrosomal PLK4 staining intensity in Tiam1 knockdown cells. (B) Quantification of PLK4 intensity at centrosomes in U2OS cells from three independent experiments from images in A [total number of cells: *n*=186 (control); *n*=175 (KD#1); *n*=165 (KD#2); *n*=170 (KD#3)]. (C) Maximal *z*-projections of HCT116 cells stained for pericentrin (magenta) as a marker of centrosomes, and endogenous PLK4 (green), showing an increase in centrosomal PLK4 staining in Tiam1 knockdown cells. (D) Quantification of PLK4 intensity at centrosomes in HCT116 cells from two independent experiments from images in C [total number of cells: *n*=237 (control); *n*=97 (KD#1); *n*=251 (KD#2); *n*=296 (KD#3)]. (E) Representative WB showing an increase in expression of exogenous myc-tagged PLK4 (as determined by immunoprecipitation of myc-tagged PLK4) in U2OS-PLK4 cells following Tiam1 knockdown, compared with control transfected cells. Doxycycline (dox) was added to induce expression of exogenous myc-tagged PLK4. α-tubulin was used as a loading control. Two-tailed, upaired *t*-test. (F) Quantification of increased PLK4 expression (as determined by immunoprecipitation of myc-tagged PLK4) from control and Tiam1 knockdown cells (*n*=3 independent experiments). Dots represent each individual experiment. PLK4 pull-down levels normalised to α-tubulin input. (G) Quantification of cells with excess centrin puncta in HCT116 cells transfected with either control siRNA or Tiam1 KD#3 siRNA, and treated with either vehicle or Centrinone (20 nM). Data are mean±s.e.m. For boxplots, boxes show 25th to 75th percentiles, whiskers show fifth to 95th percentiles (the median is marked with a line), and significance was determined using one-way ANOVA, corrected for multiple comparisons. *P<0.05; ****P<0.0001; n.s., not significant. Scale bars: 3 µm.

Next, we stained control or Tiam1-depleted U2OS cells for PLK4 and pericentrin. Using the same analysis method (Fig. S3E) we saw a significant increase in total centrosomal PLK4 intensity in cells treated with Tiam1 siRNA compared with control siRNA-treated cells (Fig. 3A, quantified in Fig. 3B). The equivalent experiment with HCT116 cells also demonstrated an increase in centrosomal PLK4 staining after Tiam1 depletion (Fig. 3C, quantified in Fig. 3D), demonstrating that this effect was conserved between cell lines.

To corroborate these IF data, we biochemically measured PLK4 levels following Tiam1 knockdown. We were unable to detect endogenous PLK4 by western blotting [WB, similar to reports from other researchers, as the cellular levels of PLK4 are very low (Holland et al., 2012b)], and therefore overexpressed myc-tagged PLK4 in U2OS cells using a doxycycline-inducible system (U2OS-PLK4). This induced centriole overduplication (Fig. S3G). We used anti-Myc beads to immunoprecipitate exogenous PLK4, and saw that its levels increased following Tiam1 knockdown (Fig. 3E, quantified in Fig 3F). Together, these experiments indicate that Tiam1 limits PLK4 levels at the centrosome, thereby constraining centriole duplication.

Given that Tiam1-depleted cells have elevated PLK4 levels and that high PLK4 drives centriole overduplication, we hypothesised that low-level PLK4 inhibition in Tiam1-depleted cells would restore their correct centriole number. Therefore, we treated HCT116 cells with the specific PLK4 inhibitor Centrinone (Wong et al., 2015) at 20 nM. Our preliminary experiments indicated that this concentration had a minimal effect on basal centriole duplication, with most control siRNA-treated cells containing centrosomes with two centrin puncta (data not shown). Tiam1 knockdown in the absence of the inhibitor led to an increase in cells with excess centrin puncta as previously observed (Fig. 3G). However, knockdown cells treated with Centrinone did not display an increase in cells with excess centrin puncta compared to control-siRNA-treated cells (Fig. 3G). These findings demonstrate that the production of extra centrioles in Tiam1-depleted cells requires increased PLK4 activity.

**Fig. 4.**
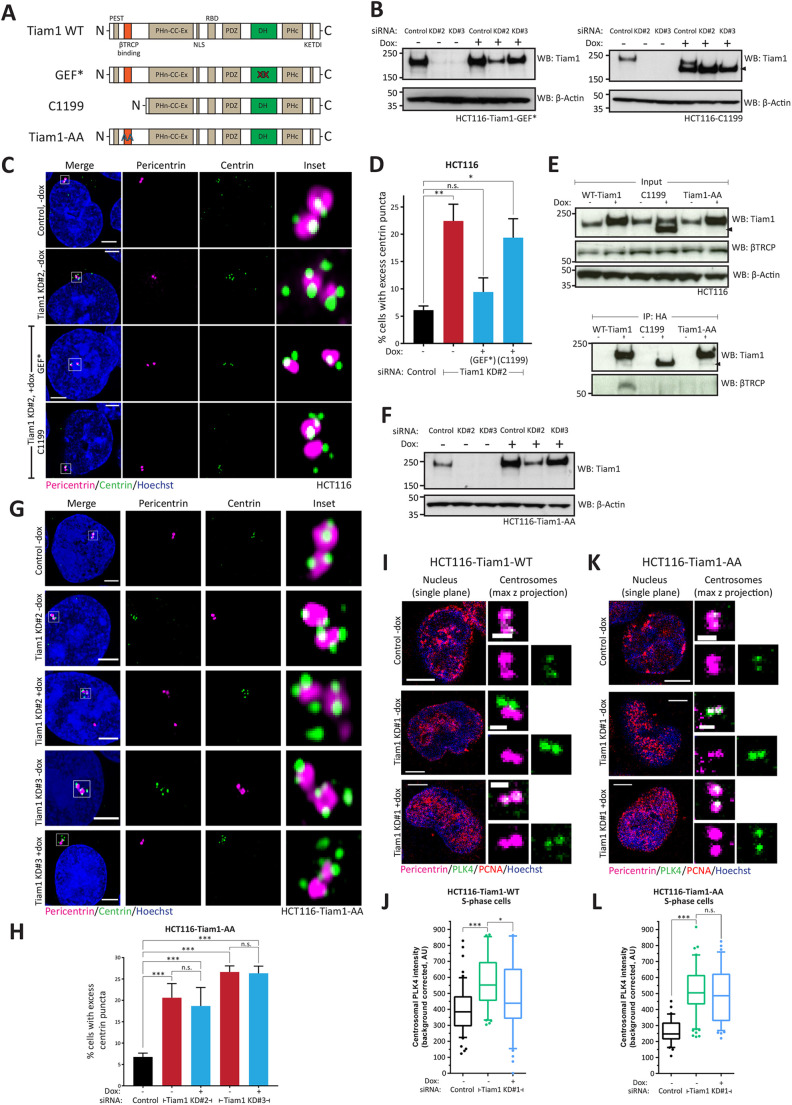
**Tiam1 requires βTRCP binding to maintain normal centriole number and centrosomal PLK4 levels.** (A) Schematic showing the domain structure of wild-type (WT) Tiam1 and the mutant versions. (B) WBs from HCT116-Tiam1-GEF* and HCT116-C1199 cells showing depletion of endogenous Tiam1 by two independent siRNAs and, following treatment with doxycycline (dox), restoration of near-endogenous levels of either a GEF-dead (GEF*) Tiam1 or an N-terminally truncated mutant (C1199, marked with an arrowhead), both of which are resistant to siRNA treatment. (C) Maximal *z*-projection confocal images of HCT116 control, Tiam1 knockdown and Tiam1 rescue cells. Cells exhibit excess centrin puncta following Tiam1 knockdown; this is rescued by expression of RNAi-resistant GEF* Tiam1, but not by expression of RNAi-resistant C1199. (D) Quantification of HCT116 cells with excess centrin puncta from rescue experiments in C, from three independent experiments. More than 50 cells were counted per condition per experiment. (E) WB from HCT116 cells showing immunoprecipitation of wild-type Tiam1, C1199 (marked with an arrowhead) and Tiam1-AA (all constructs HA-tagged and induced to express following addition of doxycycline). Only wild-type Tiam1 was able to co-immunoprecipitate endogenous βTRCP. (F) WB from HCT116-Tiam1-AA cells showing depletion of endogenous Tiam1 by two independent siRNAs and restoration of near-endogenous levels of Tiam1 following treatment with doxycycline to express the RNAi-resistant non-βTRCP-binding mutant Tiam1-AA. (G) Maximal *z*-projection confocal images of control and Tiam1-knockdown HCT116 cells, showing excess centrin puncta following Tiam1 knockdown, which is not rescued by doxycycline-induced expression of siRNA-resistant Tiam1-AA. (H) Quantification of cells with excess centrin puncta from rescue experiments in G from four independent experiments. More than 50 cells were counted per condition per experiment. (I) Single confocal planes of S-phase HCT116-Tiam1-WT cells showing centrosomal PLK4 (green). Centrosomes are marked with pericentrin (magenta), and S-phase nuclei (blue) are marked by punctate PCNA staining (red). (J) Quantification of PLK4 intensity at centrosomes in HCT116 cells from the rescue experiment shown in I [total number of cells: *n*=52 (control); *n*=48 (KD#1, without dox); *n*=40 (KD#1, plus dox)]. (K) Single confocal planes of S-phase HCT116-Tiam1-AA cells showing centrosomal PLK4 (green). Centrosomes are marked with pericentrin (magenta), and S-phase nuclei (blue) are marked by punctate PCNA staining (red). (L) Quantification of PLK4 intensity at centrosomes in HCT116 cells from the rescue experiment shown in K [total number of cells: *n*=30 (control); *n*=70 (KD#1, without dox); *n*=46 (KD#1, plus dox)]. Data are mean±s.e.m. Boxes show 25th to 75th percentiles, and whiskers show 10th to 90th percentiles (the median is marked with a line). Statistical significance was determined using one-way ANOVA, corrected for multiple comparisons (D,H), and a two-tailed, unpaired *t*-test (for comparing +/− dox cells treated with the same siRNA in H,J) . *P<0.05; **P<0.01; ***P<0.001; n.s., not significant. β-actin was used as a loading control in all blots. AU, arbitrary units. Scale bars: 3 µm (C,G); 5 µm (nuclei in I,K); 1 µm (centrosomes in I,K).

Given that wild-type Tiam1 was able to rescue centriole overduplication, as determined by changes in excess centrin puncta (Fig. 1I,J), we next performed structure-function experiments with mutant versions of Tiam1 to investigate the mechanism by which Tiam1 regulates PLK4 levels. As Tiam1 is best known for its ability to activate Rac1, we addressed whether the catalytic (GEF) activity of Tiam1 was required for correctly regulating centriole numbers. We therefore expressed a doxycycline-inducible siRNA-resistant ‘GEF-dead’ mutant (GEF*) Tiam1 (Fig. 4A) (Tolias et al., 2005) in HCT116 cells treated with either control siRNA or siRNA against endogenous Tiam1 (Fig. 4B). First, we confirmed that GEF* Tiam1 localised to centrosomes similarly to wild-type Tiam1 (Fig. S4A). Next, we saw that expression of GEF* Tiam1 significantly reduced the number of cells with excess centrin puncta following siRNA-mediated depletion of Tiam1, despite the inability of this mutant to activate Rac1 (Fig. 4C, quantified in Fig. 4D). This indicates that Tiam1-dependent Rac1 activation is not required for normal centriole duplication or its localisation to the centrosome.

We also observed that doxycycline-induced overexpression of GEF* Tiam1 in cells still expressing endogenous Tiam1 was able to significantly restrain centriole overduplication resulting from hydroxyurea treatment as measured by changes in centrin puncta, similar to expression of WT-Tiam1 (Fig. S4B,C, Fig. S2F-H). Together, these results indicate that the GEF activity of Tiam1 is dispensable with respect to the control of centriole duplication. This also suggests that this pathway is distinct from the one controlling centrosome separation during prophase and prometaphase, which does rely on Tiam1-mediated Rac1 activation (Whalley et al., 2015; Woodcock et al., 2010). To further investigate the relationship between these two centrosomal Tiam1 functions, we performed rescue experiments with Tiam1-1466A, a CDK1-phosphorylation mutant version of Tiam1 (Fig. S4D), which is unable to rescue centrosome separation in prophase despite correct localisation to centrosomes (Whalley et al., 2015). As with the GEF* mutant, expression of this mutant Tiam1 was able to restore normal centriole number after Tiam1 knockdown (Fig. S4E,F), demonstrating that this role of Tiam1 is distinct from that of Tiam1 in prophase.

As the function of Tiam1 in centriole duplication appeared to be independent of Rac1, some other domain of Tiam1 must play a role in regulating centriole duplication. Indeed, Tiam1 is a large multi-domain protein that also acts as a molecular scaffold (Marei et al., 2016). We therefore performed our rescue experiment again with a well-characterised N-terminally truncated mutant of Tiam1, C1199 (Hordijk et al., 1997) (Fig. 4A). Interestingly, doxycycline-induced expression of siRNA-resistant C1199 (Fig. 4B) was unable to significantly reduce the number of HCT116 cells with excess centrin puncta following Tiam1 knockdown (Fig. 4C, quantified in Fig. 4D). This suggests that a function performed by the N-terminus of Tiam1 is required for normal centriole duplication. Significantly, this region of Tiam1 contains a phospho-degron required for binding βTRCP (Magliozzi et al., 2014), a component of the E3-ligase complex also targeting centrosomal PLK4 for degradation during S-phase (Fig. S1A) (Guderian et al., 2010; Cunha-Ferreira et al., 2009).

We reasoned that the interaction between Tiam1 and βTRCP may be required for the ability of Tiam1 to regulate PLK4 levels at the centrosome, and that the C1199 mutant may be unable to substitute for endogenous Tiam1 because of its lack of βTRCP binding. To test this, we produced a mutant version of full-length Tiam1 (referred to as Tiam1-AA; Fig. 4A) containing two point mutations in the βTRCP phospho-degron (Magliozzi et al., 2014). We confirmed that both this mutant and C1199 were unable to bind to βTRCP by co-immunoprecipitation (Fig. 4E). We also confirmed that Tiam1-AA was able to correctly localise to the centrosome in the same way as wild-type Tiam1 (Fig. S4A), indicating that βTRCP binding was not required for centrosomal localisation of Tiam1. We then performed Tiam1 knockdown and rescue experiments, inducibly expressing the siRNA-resistant Tiam1-AA mutant to near-endogenous levels in cells depleted of Tiam1 (Fig. 4F). In both uninduced (−dox) and induced (+dox) cells there was a significant and indistinguishable increase in cells with excess centrin puncta upon endogenous Tiam1 depletion compared with control-siRNA treated cells (Fig. 4G,H), showing that expression of Tiam1-AA is unable to compensate for depletion of endogenous Tiam1. Therefore, we conclude that the regulation of normal centriole number requires the interaction between Tiam1 and βTRCP. We also performed an additional CoIP experiment that confirmed that the Tiam1 GEF* mutant is able to bind to βTRCP (Fig. S4G), supporting our hypothesis that the centriole duplication and Rac1-activation roles of Tiam1 are distinct.

If the role of centrosomal Tiam1 is to maintain appropriate levels of centrosomal PLK4 in S-phase, then expression of wild-type Tiam1 should suppress the increase in centrosomal PLK4 following Tiam1 depletion. If this role requires interaction with βTRCP, then the Tiam1-AA mutant should be unable to suppress increases in centrosomal PLK4. To test this, we stained cells with an antibody against PLK4, and co-stained with PCNA to specifically identify cells in S-phase (Fig. 4I). We then quantified centrosomal PLK4 intensity in this S-phase population by imaging on the Leica SP8 confocal microscope, using the hybrid detectors in their quantitative photon counting mode, similar to the method we used previously for quantitative image analysis (Porter et al., 2019). Using this complementary imaging modality, we again saw that Tiam1 depletion led to an increase in centrosomal PLK4 (Fig. 4I, quantified in Fig 4J; see also Fig. 3C,D). Expression of siRNA-resistant wild-type Tiam1 was able to significantly decrease the centrosomal PLK4 signal, partially rescuing the effect of Tiam1 knockdown (Fig. 4I, quantified in Fig. 4J). A second experiment demonstrated the same trend, although with lower absolute pixel intensities (Fig. S4H). Next, we tested whether the Tiam1-AA mutant was able to act in the same manner as the wild-type protein. In the absence of doxycycline, we saw that Tiam1 knockdown again increased S-phase centrosomal PLK4 intensity in HCT116-AA cells (Fig. 4K,L). However, unlike expression of wild-type Tiam1, Tiam1-AA expression did not lead to any decrease in centrosomal PLK4 intensity in knockdown cells (Fig. 4K,L; additional experiment in Fig. S4I). From these experiments, we conclude that the normal role of Tiam1 is to positively regulate the degradation of PLK4 by βTRCP, maintaining an appropriate level of S-phase PLK4 for centriole duplication, and that this function does not depend on activating Rac1.

**Fig. 5.**
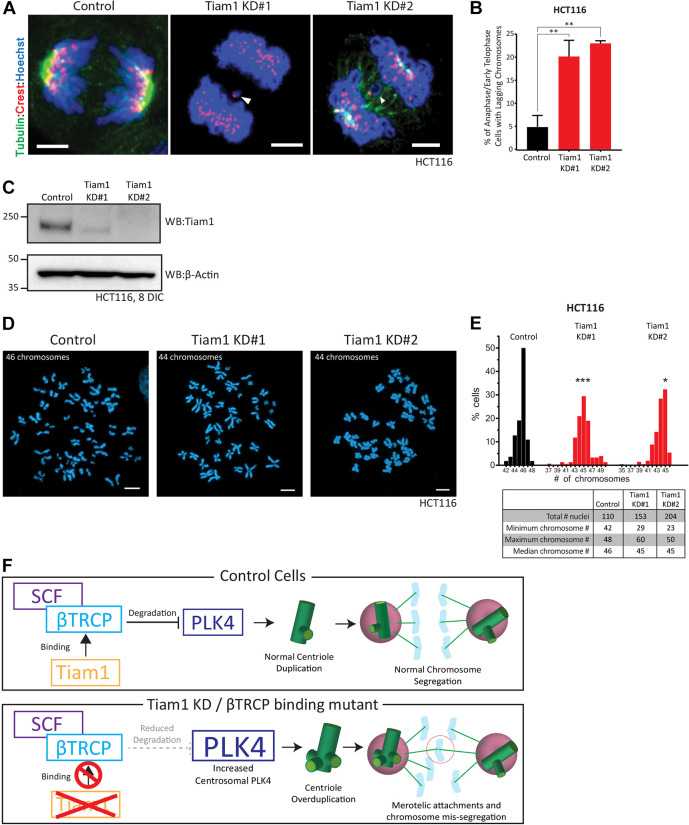
**Depletion of Tiam1 leads to lagging chromosomes at anaphase and early telophase and aneuploidy.** (A) Maximal *z*-projection confocal images of anaphase HCT116 cells. Chromosomes stained with Hoechst and marked with CREST as a centromere marker, showing lagging chromosomes following Tiam1 knockdown with two independent siRNAs. (B) Quantification of the percentage of HCT116 cells in anaphase or early telophase with lagging chromosomes following transfection with control or Tiam1 knockdown siRNAs, as in A. Data quantified from three independent experiments. At least 50 cells were quantified per condition per experiment (one-way ANOVA, corrected for multiple comparisons). (C) WB showing Tiam1 levels in HCT116 cells following 8 days growth in culture with repeated siRNA transfection. β-actin was used as a loading control. (D) Representative images of metaphase spreads from HCT116 cells treated with either control or Tiam1 siRNAs. (E) Histogram of chromosome number from control and Tiam1 knockdown HCT116 cells. Data were collected from three independent experiments. Table shows additional statistics. *P<0.05, **P<0.01, ***P<0.001 (Kruskal–Wallis test adjusted for multiple comparisons). (F) Model of how the Tiam1-βTRCP interaction affects PLK4 protein levels, centriole duplication and chromosome segregation. Scale bars: 3 µm (A); 5 µm (D).

Centriole overduplication can lead to chromosome mis-segregation, aneuploidy and CIN (Godinho and Pellman, 2014), ultimately leading to tumourigenesis (Raff and Basto, 2017; Basto et al., 2008; Levine et al., 2017). Chromosome mis-segregation can occur via merotelic attachments occurring during the formation of transient multipolar intermediates arising from centrosome amplification (Ganem et al., 2009), of which we see a small increase following Tiam1 depletion (Fig. S2D,E). Chromosome mis-segregation can also occur through an imbalance of centriole numbers at the two poles of the mitotic spindle, which leads to differences in microtubule nucleation between the poles and does not trigger the cellular error correction machinery (Cosenza et al., 2017). Interestingly, detailed analysis of mitotic U2OS cells stained with CP110 and SAS6 revealed that all the cells with excess CP110 or SAS6 puncta had asymmetric centriole numbers at the poles, suggesting that this might strongly affect chromosome segregation (data not shown). Interestingly, Tiam1 depletion, although largely suppressing tumour formation (Malliri et al., 2002, 2006), promotes malignant progression (Malliri et al., 2002, 2006), indicating a dual oncogene/tumour suppressor role for Tiam1 (Maltas et al., 2020). As CIN is considered to drive the acquisition of malignant hallmarks (Giam and Rancati, 2015; Hanahan and Weinberg, 2011), we investigated whether knockdown of Tiam1 could lead to an increase in lagging chromosomes at anaphase, a widely used readout of chromosome mis-segregation that can lead to CIN. We combined Hoechst staining with a centromere marker (CREST) to distinguish true lagging chromosomes from acentromeric chromosome fragments and chromosome bridges (Fig. 5A). In the chromosomally stable HCT116 cell line, we observed a low level of lagging chromosomes at anaphase and early telophase in control-siRNA-treated cells (Fig. 5A, quantified in Fig. 5B). Following Tiam1 knockdown, the number of cells with lagging chromosomes significantly increased (Fig. 5A,B). There was no change in the number of chromosome bridges or acentric chromosomes (data not shown), suggesting that Tiam1 knockdown affects the segregation of whole chromosomes specifically. We saw similar increases in lagging chromosomes in U2OS cells depleted for Tiam1 (Fig. S5A, quantified in Fig. S5B), indicating conservation of the role of Tiam1 in chromosome segregation.

Given this increase in lagging chromosomes, and the previously reported chromosome alignment defects arising from Tiam1 knockdown (Woodcock et al., 2010), we tested whether these would translate into an increase in aneuploidy given longer periods of Tiam1 depletion. We grew HCT116 cells for 8 days with two rounds of siRNA transfection (Fig. 5C), and prepared metaphase spreads. Chromosome number was determined by manual counting of Hoechst-stained chromosomes (representative images in Fig. 5D). Although the range of chromosome number found in control cells was relatively narrow (from 42 to 48 chromosomes), we found a much wider distribution of chromosome numbers in cells treated with Tiam1 siRNA (from 29 to 60 for Tiam1 KD#1, and from 23 to 50 for Tiam1 KD#2), indicating a significant increase in aneuploidy following Tiam1 depletion (Fig. 5E). Our data indicate that Tiam1 contributes to the control of chromosome segregation through regulating centriole duplication.

## DISCUSSION

In this study, we identify Tiam1 as a new regulator of PLK4, the master regulator of centriole duplication. PLK4 regulation involves a fine balance between a brief period of activity – sufficient to initiate procentriole formation – and ubiquitin-mediated degradation to prevent re-duplication. Too much activity leads to overduplication (Conduit et al., 2015; Guderian et al., 2010; Holland et al., 2010), too little to centriole loss (Wong et al., 2015), but the full details of this precise spatiotemporal regulation remain to be determined (Nigg and Holland, 2018). Defects in PLK4 levels can ultimately lead to defects in chromosome segregation (Ganem et al., 2009; Levine et al., 2017) and other pro-oncogenic effects, such as increased invasion (Godinho et al., 2014; Cosenza and Krämer, 2016).

Our data indicate that Tiam1 acts as a modulator of PLK4 protein levels. Depletion of Tiam1 leads to an increase in centrosomal PLK4, which can be rescued by re-expression of wild-type Tiam1, but not by Tiam1, which is unable to bind βTRCP (Tiam1-AA). Our PLK4 imaging setups using both widefield and confocal microscopy were optimised for intensity measurements; more specialist super-resolution imaging (such as in Ohta et al., 2018) may determine whether there are subtler changes to PLK4 localisation or the number of PLK4 puncta. Depletion of Tiam1 leads to an increase in centrosomes containing excess puncta of three distinct centriole markers, and a small increase in cells with centrosome amplification (see model in Fig. 5F), and overexpression of Tiam1 constrained centriole overduplication caused by hydroxyurea treatment. The increase in centrioles, as reported by centrin staining, can be rescued by expression of WT-Tiam1 and Tiam1-GEF*, but not by Tiam1 lacking βTRCP binding (C1199 or Tiam1-AA), further supporting the link between Tiam1 and βTRCP binding. Although we have yet to establish how precisely Tiam1 modulates PLK4 levels through βTRCP, in a previous publication we showed that the interaction between βTRCP and Tiam1 was necessary for the degradation of TAZ, another βTRCP target protein (Diamantopoulou et al., 2017). Given our current findings, this raises the possibility that Tiam1 is able to act as a scaffold for βTRCP more generally, directing it to its targets, or enhancing its interaction with target proteins. As Tiam1 is itself a target of βTRCP-mediated degradation, this could allow for precise temporal control of βTRCP-target degradation, as once Tiam1 itself is degraded the targeting effect would be removed. The ability of the catalytically inactive GEF* mutant of Tiam1 to rescue Tiam1 depletion and oppose hydroxyurea-induced centriole overduplication further supports the role of Tiam1 as a scaffold protein, alongside its better characterised role as a Rac1-GEF (Marei et al., 2016; Marei and Malliri, 2017). The regulation of centriole duplication by Tiam1 appears distinct from its role in driving centrosome separation during prophase (Woodcock et al., 2010), as, unlike the latter, the former does not depend on the activation of Rac1, nor does it require phosphorylation of Tiam1 at S1466. It is interesting to speculate whether these two centrosomal functions of Tiam1 are temporally segregated, and if so how. Perhaps distinct complexes of Tiam1 exist at centrosomes (including either βTRCP and PLK4 or Rac1 and PAK1/2) dictated by mutually exclusive intermolecular interactions. Alternatively, post-translational modification of Tiam1, such as by CDK1 phosphorylation (Whalley et al., 2015), might control a temporal switch between interacting partners.

Targeting the Tiam1-Rac pathway therapeutically remains a subject of ongoing investigation, due to the dramatic reduction in tumour formation following loss of Tiam1 in animal models (Malliri et al., 2006, 2002). However, those models also reveal a dual role for Tiam1. Although significantly fewer in number, tumours lacking Tiam1 are more likely to become malignant, indicating that Tiam1 deficiency promotes malignant conversion (Malliri et al., 2006, 2002).

We show that loss of Tiam1 leads to an increase in lagging chromosomes at anaphase, with ∼25% of knockdown cells presenting with lagging chromosomes. We also see centriole abnormalities in ∼25% of the population, and analysis in mitotic cells shows that this often affects one pole of the mitotic spindle, generating asymmetric centriole numbers, which are a potent driver of chromosome mis-segregation (Cosenza et al., 2017). With only a small percentage of cells displaying full centrosome amplification, further analysis (such as staining with markers of more mature centrioles and electron microscopy reconstructions of the centriole structures) will be required to better understand this relatively low conversion rate, although the increase in CP110 staining suggests that these centriolar structures are undergoing at least part of the centriolar maturation process. As both the U2OS and HCT116 cells used here have a functioning p53 pathway, it is possible that the centriole defects are triggering an apoptotic response such that cells with excess centrosomes are being lost from the population, or that a more subtle change in cell cycle progression is occurring that we have yet to detect.

We see an increase in aneuploidy itself, a potential driver of malignant conversion, which is due to a combination of the effects of centriole overduplication and centrosome amplification demonstrated here, chromosome congression defects that arise from Tiam1 depletion, as we described previously (Woodcock et al., 2010; Whalley et al., 2015), and potentially other effects following the loss of Tiam1. Together, these indicate a pathway by which Tiam1 depletion could enhance tumour progression, and highlights a need for a more detailed understanding of Tiam1 signalling to separate its pro- and anti-tumourigenic properties (Maltas et al., 2020).

## MATERIALS AND METHODS

### Cell culture

All cell lines were cultured at 37°C in a humidified incubator (5% CO_2_ atmosphere). U2OS and HCT116 cells were cultured in Dulbecco's modified Eagle Medium (DMEM) High Glucose (Gibco) supplemented with 10% fetal bovine serum (FBS, Gibco). MCF7 cells were cultured in DMEM High Glucose (Sigma-Aldrich) supplemented with 10% FBS and 1% L-Glutamine (Gibco). Cell lines were routinely tested to exclude Mycoplasma contamination and for cell line authentication (via in-house facilities).

### Generation of cell lines

Plasmids were introduced into cells either by transfection using TransIT-LT1 (Mirus), according to the manufacturer's instructions, or by retroviral transduction as described previously (Woodcock et al., 2010). For inducible overexpression, HCT116 were retrovirally transduced with pRetro-Tet-ON followed by selection with G418 (1 mg/ml, Sigma-Aldrich). pRetro-XT-based constructs were then retrovirally transduced and cells selected with puromycin (2 μg/ml, Sigma-Aldrich). For transfection of WT-Tiam1-eGFP and eGFP (control), U2OS cells were transfected using LT1 transfection reagent (Mirus), and stably selected using G418 (1 mg/ml, Sigma-Aldrich).

### Plasmids

The doxycycline-inducible PLK4 plasmid was generated by PCR of the wild-type PLK4 cDNA from Addgene plasmid 41165 using PfuUltra II Fusion HS DNA polymerase (Agilent, 600670). pcDNA Plk4(Sak) wt (Nigg HR9) was a gift from Erich Nigg (Habedanck et al., 2005). The sequence was confirmed by PCR, and digested with NotI and MluI enzymes (restriction sites introduced via the PCR primer sequences) for introduction into similarly digested pRetro-X-Tight (Takara Bio). The final insertion was confirmed by sequencing.

The doxycycline-inducible Tiam1-AA (non-βTRCP-binding) mutant was generated by Quikchange II (Agilent) site-directed mutagenesis of WT-Tiam1 mouse cDNA at S329 and S334, and successful mutagenesis confirmed by PCR. A portion of the cDNA containing the mutated region was digested with HpaI and NotI, and inserted into the existing pRetro-X-Tight-Tiam1-WT plasmid. Insertion was confirmed by PCR.

### siRNA transfection

For transfection of cells for IF staining, cells were plated at a density of 2×10^5^ onto glass coverslips (six-well dish), and reverse transfected using RNAiMax (Invitrogen) following the manufacturer's instructions. Cells were grown for 72 h before being fixed in MeOH. For Tiam1, four siRNA sequences (synthesized by Eurofins MWG) were used: Tiam1 KD#1, 5′-GAGGTTGCAGATCTGAGCA-3′; Tiam1 KD#2, 5′-GAGGUUGCAGAUCUGAGCA-3′; Tiam1 KD#3, 5′-AGAGCGCACCUACGUGAAA-3′; and Tiam1 KD#4, 5′GGTTCTGTCTGCCCAATAA3′. In all of the reported assays, a negative control siRNA was used, typically siLuc (control), 5′-CGUACGCGGAAUACUUCGA-3′, or Dharmacon siGENOME Non-Targeting siRNA #4. Four siRNA sequences were used to deplete PLK4 to test the specificity of the anti-PLK4 antibody. These were as follows: PLK4 siRNA#1, 5′-GAAAUGAACAGGUAUCUAA-3′; PLK4 siRNA#2, 5′-GAAACAUCCUUCUAUCUUG-3′; PLK4 siRNA#3, 5′-GUGGAAGACUCAAUUGAUA-3′; and PLK4 siRNA#4, 5′-GGACCUUAUUCACCAGUUA-3′ (sequences derived from Holland et al., 2012a).

### Hydroxyurea treatment

U2OS tumour cells exogenously expressing eGFP, Tiam1-WT-eGFP or Tiam1-GEF*-HA constructs were seeded to coverslips at a density of 6×104 cells/ml 24 h before treatment. Cells were treated with 4 mM hydroxyurea (Sigma-Aldrich) for 96 h. Fifty eGFP^+^ or HA^+^ cells were assayed per experiment for centriole number.

### Centrinone treatment

Following siRNA transfection and plating, cells were treated with 20 nM Centrinone (Tocris Bioscience) or vehicle control for 3 days before fixation. In preliminary experiments, higher concentrations of Centrinone led to an obvious decrease in cells with centrosomes (marked with pericentrin) and an increase in centrosomes with no centrin puncta, However, 20 nM Centrinone had relatively little effect on basal centrosome and centriole numbers (data not shown). For quantification, cells were marked as abnormal if they contained centrosomes with more than 2 centrin puncta, as in other experiments in the paper.

### Depolymerisation of the cytoplasmic microtubule network

Tiam1 was transiently depleted in U2OS cells by reverse transfection of siRNA on glass coverslips. Cells, 72 h post transfection, were transferred to pre-cooled DMEM (4°C) and incubated on ice for 30 min to depolymerise cytoplasmic microtubules.

Cells were fixed in ice-cold methanol immediately following 30 min incubation on ice. Centrioles were visualised by IF using centrin and centrosomes using pericentrin. Diffuse α-tubulin staining around the centrioles revealed depolymerisation of the microtubule network. Centriole number was quantified using the Deltavision Core microscope.

### Antibodies

Antibodies against the following were used for WB, IF and immunoprecipitation: α-tubulin (DM1A; Sigma-Aldrich, T6199 mouse; 1:2500 IF, methanol; 1:5000 WB); β-actin (Sigma-Aldrich, clone AC-15 mouse; 1:10,000 WB); γ-tubulin [Santa Cruz Biotechnology, (C-20) goat; 1:1000 IF, methanol]; centrin [Millipore, (20H5) mouse; 1:5000 IF, methanol]; CP110 (Proteintech, 12780-1-AP, Rabbit; 1:1000 IF, methanol); CREST (Europa Bioproducts, FZ90C-CS1058, human, 1:2000, methanol); HA tag (Roche Diagnostics, 3F10 rat; 1:200 IF, formaldehyde; 1:1000 WB); PCNA (Abcam, ab18197, rabbit, 1:2000 IF, methanol); pericentrin (Covance, PRB-432C; rabbit, 1:2000 IF, methanol); PLK4 (Sigma-Aldrich, clone 6H5 mouse, 1:1000 IF, methanol; 1:500 WB); SAS6 (Santa Cruz Biotechnology, sc-81431, mouse, 1:1000 IF, methanol); Tiam1 (Bethyl Laboratories, rabbit, A300-099A, 1:1000 WB); and Tiam1 (R&D Systems, AF5038, sheep 1:200, methanol).

Secondary antibodies and stains used were as follows: IgG peroxidase-conjugated anti-mouse IgG from donkey (GE Healthcare, NA931), anti-rabbit IgG from donkey (GE Healthcare, NA934); Alexa Fluor 488 chicken anti-mouse IgG (H+L) (Molecular Probes, A21200), Alexa Fluor 488 donkey anti-rat IgG (H+L) (Molecular Probes, A21208), Alexa Fluor 568 donkey anti-mouse IgG (H+L) (Molecular Probes, A10037), Alexa Fluor 647 chicken anti-rabbit IgG (H+L) (Molecular Probes, A21208) (1:500 IF); Alexa Fluor 647 donkey anti-sheep IgG (H+L) (Molecular Probes, A-21448 – used against both sheep and goat primary antibodies); Alexa Fluor 647 goat anti-human IgG (H+L) (Molecular Probes, A21445) (1:500 IF); and Hoechst 3342 (Life Technologies, H3570).

### Protein analysis

Cells were lysed in an appropriate volume of IP lysis buffer [50 mM Tris-HCl (pH 7.5), 150 mM NaCl, 1% Triton X-100 (v/v), 10% glycerol (v/v), 2 mM EDTA, 25 mM NaF and 2 mM NaH_2_PO_4_ containing 1% protease inhibitor cocktail (P8340, Sigma-Aldrich) and 1% phosphatase inhibitor cocktails 2 and 3 (P5726 and P0044, Sigma-Aldrich) added fresh] or RIPA buffer [25 mM Tris (pH 7.5), 150 mM NaCl, 0.1% SDS (v/v), 0.5% sodium deoxycholate (v/v), 1% Triton X-100, containing 1 EDTA-free protease inhibitor tablet (Roche), and 1% phosphatase inhibitor cocktails 1 and 2 (Sigma-Aldrich) added fresh] for 10 min on ice, and proteins were resolved by SDS-PAGE for WB.

For immunoprecipitation of myc-tagged PLK4, lysates were incubated with 50 μl of Myc-tag beads [A7470, Sigma-Aldrich, blocked with 5% bovine serum albumin (BSA) for 1 h at room temperature], for 2 h at 4°C with rotation. The beads were subsequently washed with lysis buffer and eluted with 2× SDS-PAGE sample buffer (Nupage, Invitrogen). Other immunoprecipitation experiments were performed as described previously (Whalley et al., 2015).

### Immunofluorescence

For IF, cells were grown on coverslips and fixed with 100% ice-cold methanol for 5 min at −20°C. Cells were washed and then blocked in 1% BSA in PBS (v/v) for 1 h, before successive incubation with primary antibodies (overnight at 4°C) and then secondary antibodies (1 h at room temperature). Coverslips were mounted onto glass slides using Fluromount-G (Southern Biotech) along with Hoechst 33342 (1:5000) for nuclear staining, or a droplet of ProLong Gold anti-fade reagent containing the DNA stain DAPI. Staining of endogenous Tiam1 was performed using a protocol described by Whalley et al. (2015), which was shown to specifically detect centrosomal Tiam1.

### Metaphase spreads

HCT116 cells were grown for 8 days, with an initial siRNA transfection followed by two subsequent trypsinisation, replating and transfections (total of three rounds of siRNA transfection). Cells were treated with 150 nM nocodazole (M1402, Sigma-Aldrich) for 4 h to arrest cells in mitosis. Mitotic cells were collected by a mitotic shake off and harvested, followed by resuspension in 3 ml pre-warmed hypotonic buffer [40% RPMI (R8758, Sigma-Aldrich), 60% ddH_2_O] for 20 min. Cells were fixed by repeated addition of Carnoy's fixative (3:1 v/v solution of methanol:glacial acetic acid), centrifugation and aspiration before final collection in 100% glacial acetic acid. Cells were dropped onto precooled (4°C) wet slides from a height of 40-50 cm, left to dry and stained with Hoechst. Images were taken using a Zeiss AiryScan confocal microscope, and chromosome number was determined by manual counting using ImageJ.

### Microscopy

Centrosome and centriole images were acquired using a Zeiss Observer microscope equipped with a Zeiss LSM 880 scan head with the AiryScan detector, with argon laser 458, 488, 514 nm (Lasos, Jena, Germany). Diode 405-30 (Lasos), DPSS 561-10 (Lasos) and HeNe 633 nm (Lasos) were used for illumination along with a Plan-Apochromat 40×/1.4 Oil (Zeiss) objective lens. Centriole, lagging chromosome and metaphase spread images were acquired using the ‘Fast’ AiryScan mode. All equipment control, acquisition and processing of AiryScan images was performed with Zen Black (Zeiss). All images were processed using ImageJ. Images in the same figure panel stained with the same antibody are all set to the same minimum and maximum brightness for comparison of localisation and intensity.

PLK4 images in Fig. 3 and Fig. S3 were captured using a Zeiss Axiovert 200M microscope (Solent Scientific). The system uses an Andor iXon 888 camera and a 300 W xenon light source was used for fluorescence illumination with a variety of ET-Sedat filters (406, 488, 568, 647 nm). The system uses the Metamorph software to capture and process images. Images were taken using a 100× oil lens. PLK4 images for the rescue experiments in Fig. 4 were captured using an inverted Leica TCS SP8 confocal microscope equipped with PMT and Hybrid (HyD) detectors, with the tunable white light laser for Alexa Fluor 488, Alexa Fluor 555 and Alexa Fluor 647, and 405 nm UV laser for Hoechst 33342 with a ×100 1.4 NA oil immersion objective (Leica). The Hybrid detectors were used in their proprietary photon counting mode to capture PLK4 intensity in a more quantitative fashion. Images were captured using LAS AF (3.0.1) Leica software.

IF images of mitotic U2OS cells in Fig. 2A, as well as in cold-treated U2OS cells, were captured using the Deltavision Core system [based on an Olympus IX71 microscope; fluorescence is achieved using a 300 W xenon light source with a variety of Sedat filter sets (406, 488, 568, 647 nm) and the attached Roper Cascade 512B camera; images were taken using 100×/60× oil lenses]. The Deltavision core system uses softWorx to capture and process images.

### Centriole counts

Widefield or confocal *z*-stacks of cells stained for centrin, CP110, SAS6 and pericentrin were used to determine centriole number. Unless otherwise stated, cells analysed are from an asynchronous population that includes interphase and mitotic cells. Planes were taken at the minimum distance recommended by the system (typically either 200 nm or 140 nm, depending on the system). Centriole counts were conducted manually using ImageJ, with any cell containing at least one centrosome (marked with pericentrin or γ-tubulin) that was associated with three or more puncta of centrin or CP110 (or two or more puncta of SAS6) counted as having excess puncta. Puncta were only counted if they were associated with pericentrin or γ-tubulin staining as markers of pericentriolar material. Also included were cells with more than two distinct centrosomes, as these were considered to have arisen from earlier centriole overduplication events (although these typically accounted for only 2-4% of abnormal cells).

### Quantification of centrosomal PLK4 intensity

For images in Fig. 3 and Fig. S3, maximal projections of widefield microscopy image stacks were generated using ImageJ. Two square regions of interest of fixed size were drawn: the first encompassing the centrosome area as marked with pericentrin (35px^2^) (a) and a second larger region of interest, centred on this first box (55px^2^) (b). The formula a−((b−a)(55^2^/35^2^)) was used to calculate the intensity of the PLK4 signal at the centrosome, adjusted for background intensity surrounding the centrosome. Images were acquired from an asynchronous culture; intensity measurements were only performed on interphase cells. For images in Fig. 4, the same approach was taken, and quantification was performed using the single confocal plane containing their brightest centrosome-associated PLK4 signal for each S-phase cell (marked with highly punctate PCNA staining).

### Statistical analysis

Appropriate statistical tests were chosen to minimise type I error associated with significance values. Statistical differences between data were analysed using Prism (GraphPad Software) with an appropriate post-hoc multiple comparisons test. Tests are specified in figure legends.

## Supplementary Material

Supplementary information

Reviewer comments

## References

[JCS252502C1] Arquint, C. and Nigg, E. A. (2016). The PLK4-STIL-SAS-6 module at the core of centriole duplication. *Biochem. Soc. Trans.* 44, 1253-1263. 10.1042/BST20160116 27911707 PMC5095913

[JCS252502C2] Balczon, R., Bao, L., Zimmer, W. E., Brown, K., Zinkowski, R. P. and Brinkley, B. R. (1995). Dissociation of centrosome replication events from cycles of DNA synthesis and mitotic division in hydroxyurea-arrested Chinese hamster ovary cells. *J. Cell Biol.* 130, 105-115. 10.1083/jcb.130.1.105 7790366 PMC2120504

[JCS252502C3] Banterle, N. and Gönczy, P. (2017). Centriole biogenesis: from identifying the characters to understanding the plot. *Annu. Rev. Cell Dev. Biol.* 33, 23-49. 10.1146/annurev-cellbio-100616-060454 28813178

[JCS252502C4] Bärenz, F., Mayilo, D. and Gruss, O. J. (2011). Centriolar satellites: busy orbits around the centrosome. *Eur. J. Cell Biol.* 90, 983-989. 10.1016/j.ejcb.2011.07.007 21945726

[JCS252502C5] Basto, R., Brunk, K., Vinadogrova, T., Peel, N., Franz, A., Khodjakov, A. and Raff, J. W. (2008). Centrosome amplification can initiate tumorigenesis in flies. *Cell* 133, 1032-1042. 10.1016/j.cell.2008.05.039 18555779 PMC2653712

[JCS252502C6] Bornens, M. (2002). Centrosome composition and microtubule anchoring mechanisms. *Curr. Opin. Cell Biol.* 14, 25-34. 10.1016/S0955-0674(01)00290-3 11792541

[JCS252502C7] Chen, Z., Indjeian, V. B., Mcmanus, M., Wang, L. and Dynlacht, B. D. (2002). CP110, a cell cycle-dependent CDK substrate, regulates centrosome duplication in human cells. *Dev. Cell* 3, 339-350. 10.1016/S1534-5807(02)00258-7 12361598

[JCS252502C8] Conduit, P. T., Wainman, A. and Raff, J. W. (2015). Centrosome function and assembly in animal cells. *Nat. Rev. Mol. Cell Biol.* 16, 611-624. 10.1038/nrm4062 26373263

[JCS252502C9] Cosenza, M. R. and Krämer, A. (2016). Centrosome amplification, chromosomal instability and cancer: mechanistic, clinical and therapeutic issues. *Chromosome Res.* 24, 105-126. 10.1007/s10577-015-9505-5 26645976

[JCS252502C10] Cosenza, M. R., Cazzola, A., Rossberg, A., Schieber, N. L., Konotop, G., Bausch, E., Slynko, A., Holland-Letz, T., Raab, M. S., Dubash, T. et al. (2017). Asymmetric centriole numbers at spindle poles cause chromosome missegregation in cancer. *Cell Rep.* 20, 1906-1920. 10.1016/j.celrep.2017.08.005 28834753

[JCS252502C11] Cunha-Ferreira, I., Rodrigues-Martins, A., Bento, I., Riparbelli, M., Zhang, W., Laue, E., Callaini, G., Glover, D. M. and Bettencourt-Dias, M. (2009). The SCF/Slimb ubiquitin ligase limits centrosome amplification through degradation of SAK/PLK4. *Curr. Biol.* 19, 43-49. 10.1016/j.cub.2008.11.037 19084407

[JCS252502C12] Diamantopoulou, Z., White, G., Fadlullah, M. Z. H., Dreger, M., Pickering, K., Maltas, J., Ashton, G., MacLeod, R., Baillie, G. S., Kouskoff, V. et al. (2017). TIAM1 antagonizes TAZ/YAP both in the destruction complex in the cytoplasm and in the nucleus to inhibit invasion of intestinal epithelial cells. *Cancer Cell* 31, 621-634.e6. 10.1016/j.ccell.2017.03.007 28416184 PMC5425402

[JCS252502C13] Doxsey, S. J., Stein, P., Evans, L., Calarco, P. D. and Kirschner, M. (1994). pericentrin, a highly conserved centrosome protein involved in microtubule organization. *Cell* 76, 639-650. 10.1016/0092-8674(94)90504-5 8124707

[JCS252502C14] Ganem, N. J., Godinho, S. A. and Pellman, D. (2009). A mechanism linking extra centrosomes to chromosomal instability. *Nature* 460, 278-282. 10.1038/nature08136 19506557 PMC2743290

[JCS252502C15] Giam, M. and Rancati, G. (2015). Aneuploidy and chromosomal instability in cancer: a jackpot to chaos. *Cell Div.* 10, 3. 10.1186/s13008-015-0009-7 26015801 PMC4443636

[JCS252502C16] Godinho, S. A. and Pellman, D. (2014). Causes and consequences of centrosome abnormalities in cancer. *Philos. Trans. R. Soc. Lond. B Biol. Sci.* 369, 20130467. 10.1098/rstb.2013.0467 25047621 PMC4113111

[JCS252502C17] Godinho, S. A., Picone, R., Burute, M., Dagher, R., Su, Y., Leung, C. T., Polyak, K., Brugge, J. S., Théry, M. and Pellman, D. (2014). Oncogene-like induction of cellular invasion from centrosome amplification. *Nature* 510, 167-171. 10.1038/nature13277 24739973 PMC4061398

[JCS252502C18] Guderian, G., Westendorf, J., Uldschmid, A. and Nigg, E. A. (2010). Plk4 trans-autophosphorylation regulates centriole number by controlling betaTrCP-mediated degradation. *J. Cell Sci.* 123, 2163-2169. 10.1242/jcs.068502 20516151

[JCS252502C19] Habedanck, R., Stierhof, Y.-D., Wilkinson, C. J. and Nigg, E. A. (2005). The Polo kinase Plk4 functions in centriole duplication. *Nat. Cell Biol.* 7, 1140-1146. 10.1038/ncb1320 16244668

[JCS252502C20] Hanahan, D. and Weinberg, R. A. (2011). Hallmarks of cancer: the next generation. *Cell* 144, 646-674. 10.1016/j.cell.2011.02.013 21376230

[JCS252502C21] Holland, A. J., Lan, W., Niessen, S., Hoover, H. and Cleveland, D. W. (2010). Polo-like kinase 4 kinase activity limits centrosome overduplication by autoregulating its own stability. *J. Cell Biol.* 188, 191-198. 10.1083/jcb.200911102 20100909 PMC2813471

[JCS252502C22] Holland, A. J., Fachinetti, D., DA Cruz, S., Zhu, Q., Vitre, B., Lince-Faria, M., Chen, D., Parish, N., Verma, I. M., Bettencourt-Dias, M. et al. (2012a). Polo-like kinase 4 controls centriole duplication but does not directly regulate cytokinesis. *Mol. Biol. Cell* 23, 1838-1845. 10.1091/mbc.e11-12-1043 22456511 PMC3350549

[JCS252502C23] Holland, A. J., Fachinetti, D., Zhu, Q., Bauer, M., Verma, I. M., Nigg, E. A. and Cleveland, D. W. (2012b). The autoregulated instability of Polo-like kinase 4 limits centrosome duplication to once per cell cycle. *Genes Dev.* 26, 2684-2689. 10.1101/gad.207027.112 23249732 PMC3533073

[JCS252502C24] Hordijk, P. L., ten Klooster, J. P., van der Kammen, R. A., Michiels, F., Oomen, L. C. and Collard, J. G. (1997). Inhibition of invasion of epithelial cells by Tiam1-Rac signaling. *Science* 278, 1464-1466. 10.1126/science.278.5342.1464 9367959

[JCS252502C25] Kleylein-Sohn, J., Westendorf, J., Le Clech, M., Habedanck, R., Stierhof, Y.-D. and Nigg, E. A. (2007). Plk4-induced centriole biogenesis in human cells. *Dev. Cell* 13, 190-202. 10.1016/j.devcel.2007.07.002 17681131

[JCS252502C26] Kubo, A., Sasaki, H., Yuba-Kubo, A., Tsukita, S. and Shiina, N. (1999). Centriolar satellites: molecular characterization, ATP-dependent movement toward centrioles and possible involvement in ciliogenesis. *J. Cell Biol.* 147, 969-980. 10.1083/jcb.147.5.969 10579718 PMC2169353

[JCS252502C27] Leidel, S., Delattre, M., Cerutti, L., Baumer, K. and Göczy, P. (2005). SAS-6 defines a protein family required for centrosome duplication in C. elegans and in human cells. *Nat. Cell Biol.* 7, 115-125. 10.1038/ncb1220 15665853

[JCS252502C28] Levine, M. S., Bakker, B., Boeckx, B., Moyett, J., Lu, J., Vitre, B., Spierings, D. C., Lansdorp, P. M., Cleveland, D. W., Lambrechts, D. et al. (2017). Centrosome amplification is sufficient to promote spontaneous tumorigenesis in mammals. *Dev. Cell* 40, 313-322.e5. 10.1016/j.devcel.2016.12.022 28132847 PMC5296221

[JCS252502C29] Lončarek, J., Hergert, P. and Khodjakov, A. (2010). Centriole reduplication during prolonged interphase requires procentriole maturation governed by Plk1. *Curr. Biol.* 20, 1277-1282. 10.1016/j.cub.2010.05.050 20656208 PMC2911792

[JCS252502C30] Mack, N. A., Porter, A. P., Whalley, H. J., Schwarz, J. P., Jones, R. C., Khaja, A. S. S., Bjartell, A., Anderson, K. I. and Malliri, A. (2012). beta2-syntrophin and Par-3 promote an apicobasal Rac activity gradient at cell-cell junctions by differentially regulating Tiam1 activity. *Nat. Cell Biol.* 14, 1169-1180. 10.1038/ncb2608 23103911 PMC3498067

[JCS252502C31] Magliozzi, R., Kim, J., Low, T. Y., Heck, A. J. R. and Guardavaccaro, D. (2014). Degradation of Tiam1 by casein kinase 1 and the SCFbetaTrCP ubiquitin ligase controls the duration of mTOR-S6K signaling. *J. Biol. Chem.* 289, 27400-27409. 10.1074/jbc.M114.575571 25124033 PMC4183780

[JCS252502C32] Malliri, A., van der Kammen, R. A., Clark, K., van der Valk, M., Michiels, F. and Collard, J. G. (2002). Mice deficient in the Rac activator Tiam1 are resistant to Ras-induced skin tumours. *Nature* 417, 867-871. 10.1038/nature00848 12075356

[JCS252502C33] Malliri, A., van Es, S., Huveneers, S. and Collard, J. G. (2004). The Rac exchange factor Tiam1 is required for the establishment and maintenance of cadherin-based adhesions. *J. Biol. Chem.* 279, 30092-30098. 10.1074/jbc.M401192200 15138270

[JCS252502C34] Malliri, A., Rygiel, T. P., van der Kammen, R. A., Song, J.-Y., Engers, R., Hurlstone, A. F. L., Clevers, H. and Collard, J. G. (2006). The rac activator Tiam1 is a Wnt-responsive gene that modifies intestinal tumor development. *J. Biol. Chem.* 281, 543-548. 10.1074/jbc.M507582200 16249175

[JCS252502C35] Maltas, J., Reed, H., Porter, A. and Malliri, A. (2020). Mechanisms and consequences of dysregulation of the Tiam family of Rac activators in disease. *Biochem. Soc. Trans.* 48, 2703-2719. 10.1042/BST20200481 33200195

[JCS252502C36] Marei, H. and Malliri, A. (2017). GEFs: Dual regulation of Rac1 signaling. *Small GTPases* 8, 90-99. 10.1080/21541248.2016.1202635 27314616 PMC5464116

[JCS252502C37] Marei, H., Carpy, A., Woroniuk, A., Vennin, C., White, G., Timpson, P., Macek, B. and Malliri, A. (2016). Differential Rac1 signalling by guanine nucleotide exchange factors implicates FLII in regulating Rac1-driven cell migration. *Nat. Commun.* 7, 10664. 10.1038/ncomms10664 26887924 PMC4759627

[JCS252502C38] Marteil, G., Guerrero, A., Vieira, A. F., de Almeida, B. P., Machado, P., Mendonça, S., Mesquita, M., Villarreal, B., Fonseca, I., Francia, M. E. et al. (2018). Over-elongation of centrioles in cancer promotes centriole amplification and chromosome missegregation. *Nat. Commun.* 9, 1258. 10.1038/s41467-018-03641-x 29593297 PMC5871873

[JCS252502C39] Moritz, M., Braunfeld, M. B., Guénebaut, V., Heuser, J. and Agard, D. A. (2000). Structure of the γ-tubulin ring complex: a template for microtubule nucleation. *Nat. Cell Biol.* 2, 365-370. 10.1038/35014058 10854328

[JCS252502C40] Nigg, E. A. and Holland, A. J. (2018). Once and only once: mechanisms of centriole duplication and their deregulation in disease. *Nat. Rev. Mol. Cell Biol.* 19, 297-312. 10.1038/nrm.2017.127 29363672 PMC5969912

[JCS252502C41] Nigg, E. A., Čajánek, L. and Arquint, C. (2014). The centrosome duplication cycle in health and disease. *FEBS Lett.* 588, 2366-2372. 10.1016/j.febslet.2014.06.030 24951839

[JCS252502C42] Ohta, M., Watanabe, K., Ashikawa, T., Nozaki, Y., Yoshiba, S., Kimura, A. and Kitagawa, D. (2018). Bimodal binding of STIL to Plk4 controls proper centriole copy number. *Cell Rep.* 23, 3160-3169.e4. 10.1016/j.celrep.2018.05.030 29898389

[JCS252502C43] Porter, A. P., White, G. R. M., Mack, N. A. and Malliri, A. (2019). The interaction between CASK and the tumour suppressor Dlg1 regulates mitotic spindle orientation in mammalian epithelia. *J. Cell Sci.* 132, jcs230086. 10.1242/jcs.230086 31289196 PMC6679578

[JCS252502C44] Raff, J. W. and Basto, R. (2017). Centrosome amplification and cancer: a question of sufficiency. *Dev. Cell* 40, 217-218. 10.1016/j.devcel.2017.01.009 28171744

[JCS252502C45] Schönenberger, F., Deutzmann, A., Ferrando-May, E. and Merhof, D. (2015). Discrimination of cell cycle phases in PCNA-immunolabeled cells. *BMC Bioinformatics* 16, 180. 10.1186/s12859-015-0618-9 26022740 PMC4448323

[JCS252502C46] Tolias, K. F., Bikoff, J. B., Burette, A., Paradis, S., Harrar, D., Tavazoie, S., Weinberg, R. J. and Greenberg, M. E. (2005). The Rac1-GEF Tiam1 couples the NMDA receptor to the activity-dependent development of dendritic arbors and spines. *Neuron* 45, 525-538. 10.1016/j.neuron.2005.01.024 15721239

[JCS252502C47] Tsang, W. Y., Spektor, A., Vijayakumar, S., Bista, B. R., Li, J., Sanchez, I., Duensing, S. and Dynlacht, B. D. (2009). Cep76, a centrosomal protein that specifically restrains centriole reduplication. *Dev. Cell* 16, 649-660. 10.1016/j.devcel.2009.03.004 19460342 PMC4062978

[JCS252502C48] Vaughan, L., Tan, C.-T., Chapman, A., Nonaka, D., Mack, N. A., Smith, D., Booton, R., Hurlstone, A. F. L. and Malliri, A. (2014). HUWE1 Ubiquitylates and degrades the RAC activator TIAM1 promoting cell-cell adhesion disassembly, migration, and invasion. *Cell Rep.* 10, 88-102. 10.1016/j.celrep.2014.12.012 25543140 PMC4542307

[JCS252502C49] Whalley, H. J., Porter, A. P., Diamantopoulou, Z., White, G. R. M., Castaneda-Saucedo, E. and Malliri, A. (2015). Cdk1 phosphorylates the Rac activator Tiam1 to activate centrosomal Pak and promote mitotic spindle formation. *Nat. Commun.* 6, 7437. 10.1038/ncomms8437 26078008 PMC4490568

[JCS252502C50] Wong, Y. L., Anzola, J. V., Davis, R. L., Yoon, M., Motamedi, A., Kroll, A., Seo, C. P., Hsia, J. E., Kim, S. K., Mitchell, J. W. et al. (2015). Reversible centriole depletion with an inhibitor of Polo-like kinase 4. *Science* 348, 1155-1160. 10.1126/science.aaa5111 25931445 PMC4764081

[JCS252502C51] Woodcock, S. A., Rushton, H. J., Castañeda-Saucedo, E., Myant, K., White, G. R. M. S., Blyth, K., Sansom, O. J. and Malliri, A. (2010). Tiam1-Rac signaling counteracts Eg5 during bipolar spindle assembly to facilitate chromosome congression. *Curr. Biol.* 20, 669-675. 10.1016/j.cub.2010.02.033 20346677 PMC2989435

