# Supplementary material for: The RAC1 activator Tiam1 regulates centriole duplication through controlling PLK4 levels
Source: J Cell Sci. 2021 Apr 15;134(7):jcs252502. doi: 10.1242/jcs.252502 (PMC8075378; doi:10.1242/jcs.252502)
Supplement: Supplementary information [file joces-134-252502-s1.pdf]

Supplementary Figure 1

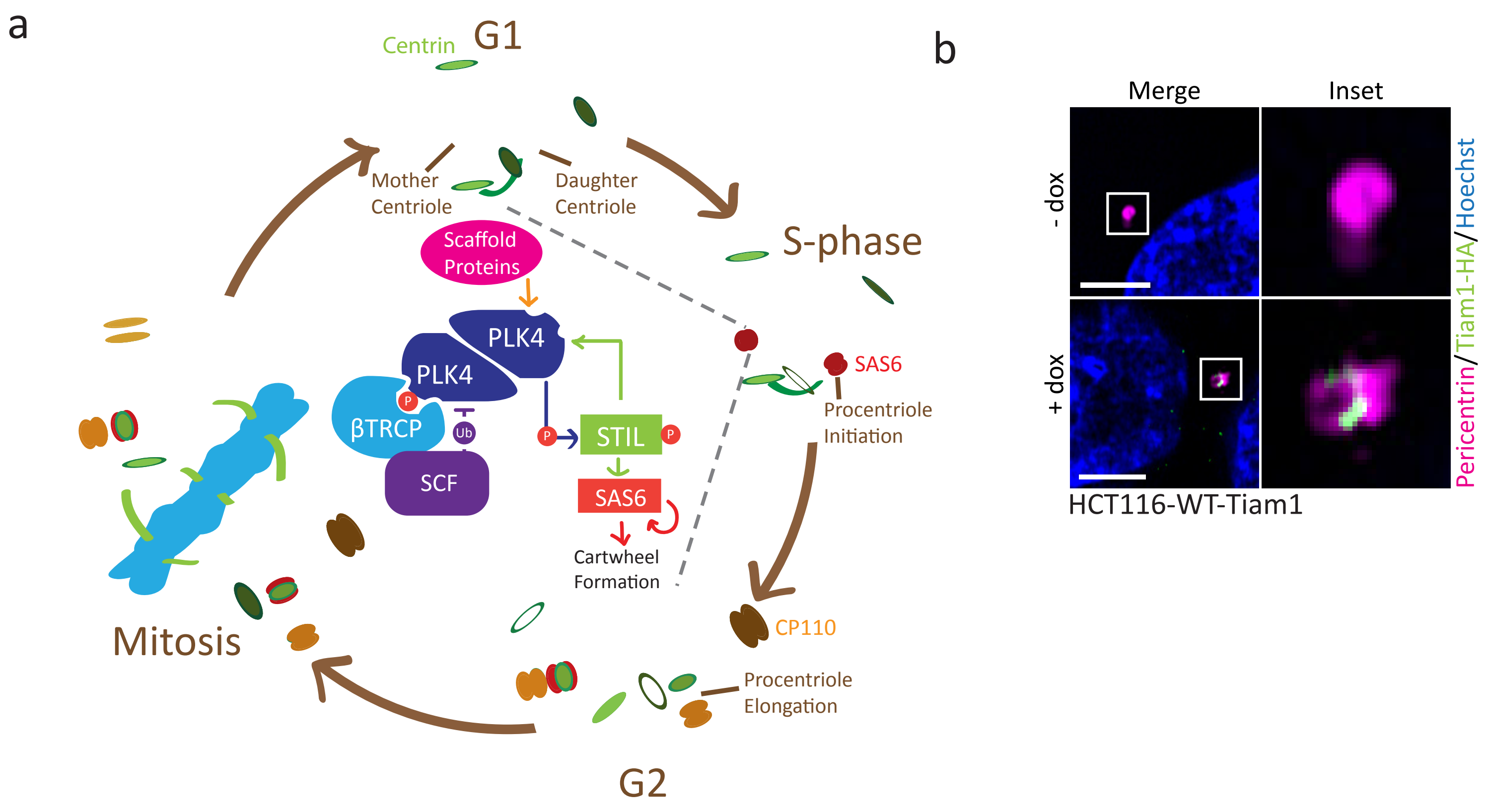

Figure S1

a) Schematic of the centriole duplication cycle. b) Single confocal z-planes of HCT116-Tiam1-WT cells showing centrosomal localisation of exogenous Tiam1 (as detected with an antibody against the HA tag on WT-Tiam1) following doxycycline (+dox) treatment.

Supplementary Figure 2

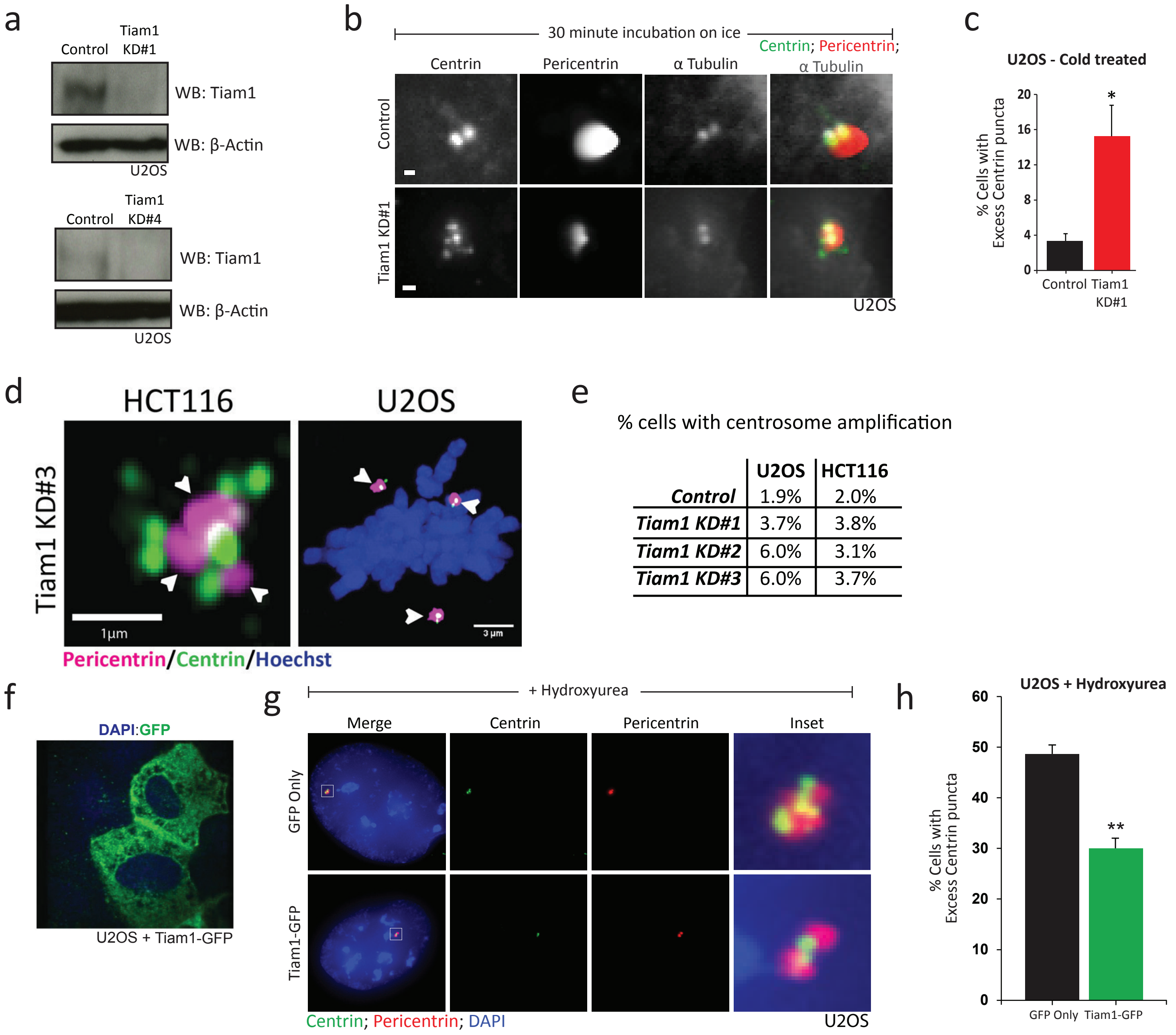

	U2OS	HCT116
Control	1.9%	2.0%
Tiam1 KD#1	3.7%	3.8%
Tiam1 KD#2	6.0%	3.1%
Tiam1 KD#3	6.0%	3.7%

Figure S2

a) Western blots showing knockdown of Tiam1 in U2OS cells following treatment with either Tiam1 KD#1 or Tiam1 KD#4 siRNAs. b) Deltavision images of centrosomes in U2OS cells (marked with Pericentrin), showing stable Centrin puncta in both control and Tiam1 knockdown cells, following cold treatment (see Methods). Scale bars show 1μm. c) Quantification of the percentage of cells with excess Centrin puncta in control and Tiam1 knockdown cells following cold treatment as in (b), from three independent experiments. ≥70 cells were quantified per experimental replicate, paired t-test. d) Representative confocal images showing HCT116 and U2OS cells with centrosome amplification (marked with arrowheads) following Tiam1 knockdown. Centrosomes are marked with Pericentrin (magenta) and Centrin (green). Scale bars are 1μm (HCT116) and 3μm (U2OS). e) Table showing increase in percentage of U2OS and HCT116 cells with excess centrosomes following 3 days of siRNA transfection. Collected from data from experiments in Figure 1. f) U2OS cells expressing Tiam1-WT-eGFP. g) Representative images of U2OS cells treated with hydroxurea and transiently transfected with either a GFP-only control plasmid or a Tiam1-WT-GFP plasmid, stained with Centrin (green) and Pericentrin (red). h) Quantification of cells with excess Centrin puncta in eGFP control and Tiam1-WT-eGFP U2OS cells after treatment with hydroxyurea (50 GFP positive cells quantified per experimental replicate; N=3, paired t-test). All bars show mean +/- SEM. *$p<0.05$ **$p<0.01$ β-Actin was used as a loading control in all blots.

Journal of Cell Science • Supplementary information

Supplementary Figure 3

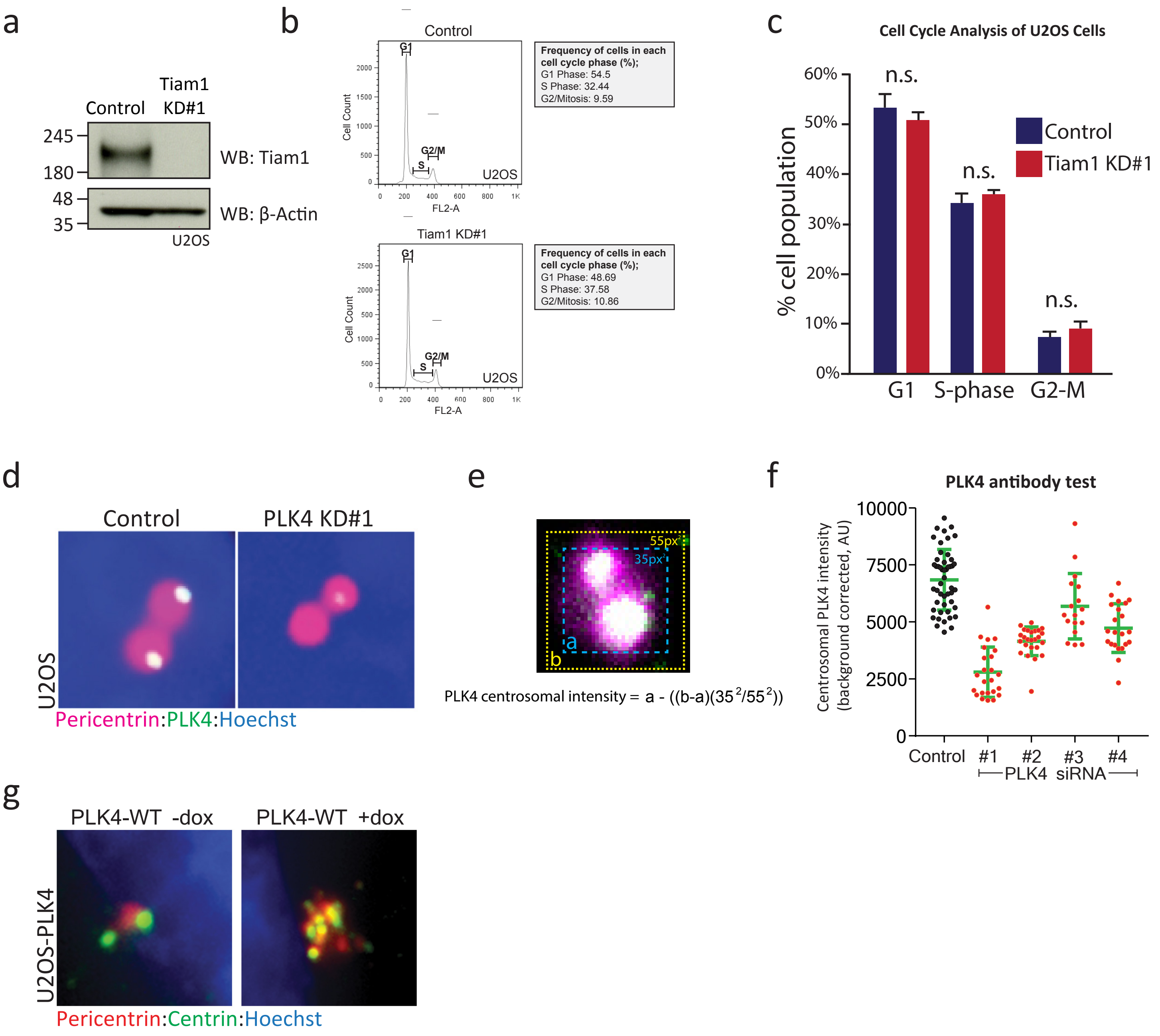

Figure S3

a) Western blot showing Tiam1 knockdown in U2OS cells. β-Actin was used as a loading control. b) Representative FACS plots from control and Tiam1 KD#1 U2OS cells. c) Summary of FACS analysis of cell cycle data from U2OS cells showing no change in cell cycle progression following Tiam1 knockdown, compared with control cells. Taken from three independent FACS experiments. d) Representative images of U2OS centrosomes (marked with Pericentrin, magenta), showing PLK4 staining (green) at centrosomes in control cells, and decrease in staining in PLK4-knockdown cells. e) Schematic of centrosomal PLK4 intensity quantification. f) Quantification of centrosomal PLK4 intensity from U2OS cells treated with a panel of siRNAs targeting PLK4. g) Images of U2OS cells stained with Pericentrin (red, marking centrosomes) and Centrin (green) showing an increase in centrosome number and Centrin puncta after treatment with doxycycline (+dox) to induce expression of WT-PLK4. t-tests comparing control and Tiam1 KD#1 for each cell cycle phase. n.s. = not significant

Journal of Cell Science • Supplementary information

Supplementary Figure 4

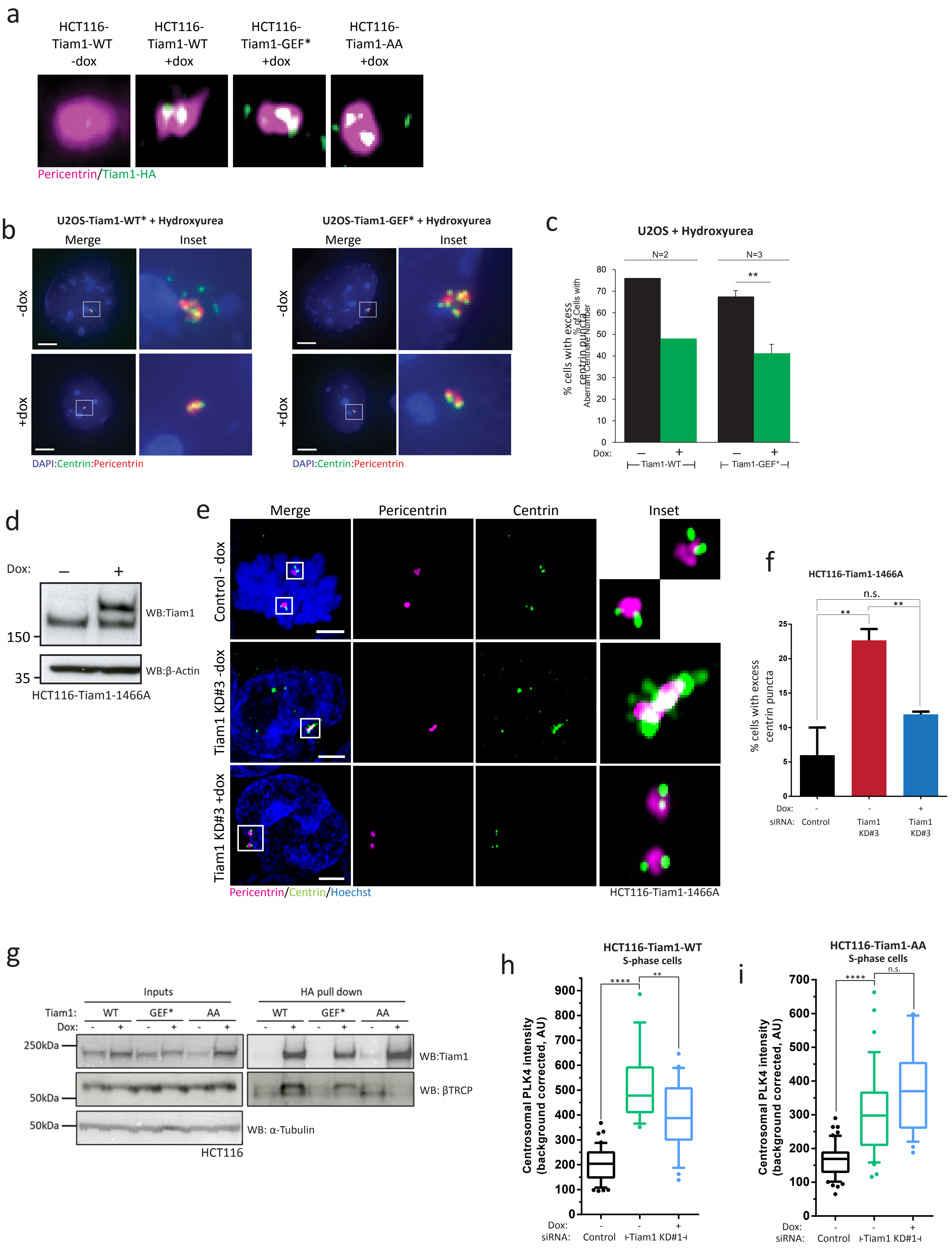

Journal of Cell Science • Supplementary information

Figure S4

a) AiryScan confocal images of centrosomes (marked with Pericentrin, magenta) of HCT116 cells, showing localization to the centrosome of exogenous WT, GEF* and AA-mutant Tiam1 (expressed following the addition of doxycycline, + dox and marked with an antibody against the HA-tag, green.) b) Deltavision images of U2OS-Tiam1-WT and U2OS-Tiam1-GEF* cells following treatment with hydroxyurea and induction of either Tiam1-WT or Tiam1-GEF* following the addition of doxycycline (dox), showing excess Centrin puncta (-dox) and normal Centrin puncta (+dox). c) Quantification of cells with excess Centrin puncta in Tiam1-WT and Tiam1-GEF* U2OS cells after treatment with hydroxyurea from experiments as in (b) (50 cells quantified per experimental replicate; N=2 (Tiam1-WT) and N=3 (Tiam1-GEF*), paired t-test). d) Western blot showing expression of Tiam1-1466A in HCT116 following treatment with doxycycline (Dox). β-Actin was used as a loading control. e) Maximal z-projection confocal images of HCT116 cells treated with either control or Tiam1 KD#3 siRNA, showing an increase in Centrin puncta following Tiam1 knockdown, which is rescued by expression of Tiam1-1466A following doxycycline (Dox) treatment. f) Quantification of HCT116 cells with excess Centrin puncta from cells as in (e) from 3 independent experiments; more than 50 cells counted per condition per experiment (one-way ANOVA, corrected for multiple comparisons). g) Western blot from HCT116 cells showing immunoprecipitation of wild-type (WT) Tiam1, Tiam1-GEF* and Tiam1-AA [all constructs HA-tagged and induced to express following addition of Doxycycline (Dox)]; Tiam1-GEF* is able to co-immunoprecipitate endogenous βTRCP, as does Tiam1-WT, but not Tiam1-AA. α-Tubulin was used as a loading control. h) Quantification of PLK4 intensity at centrosomes in HCT116-WT cells from a second rescue experiment (see also Figure 4i, j) [total number of cells: n=43 (WT, control), n=15 (WT, KD#1, -dox), n=27 (WT, KD#1, plus dox). Box shows 25th to 75th percentiles, whiskers show 10th to 90th percentiles; median is marked with a line, t-test. i) Quantification of PLK4 intensity at centrosomes in HCT116-AA cells from a second rescue experiment (see also Figure 4k, l) n=26 (AA, control), n=51 (AA, KD#1, -dox), n=36 (AA, KD#1, plus dox)]. Box shows 25th to 75th percentiles, whiskers show 10th to 90th percentiles; median is marked with a line, t-test. **** p<0.0001 ** p<0.01 n.s. = not significant; error bars show S.E.M. Scale bars show 3µm.

Journal of Cell Science • Supplementary information

Supplementary Figure 5

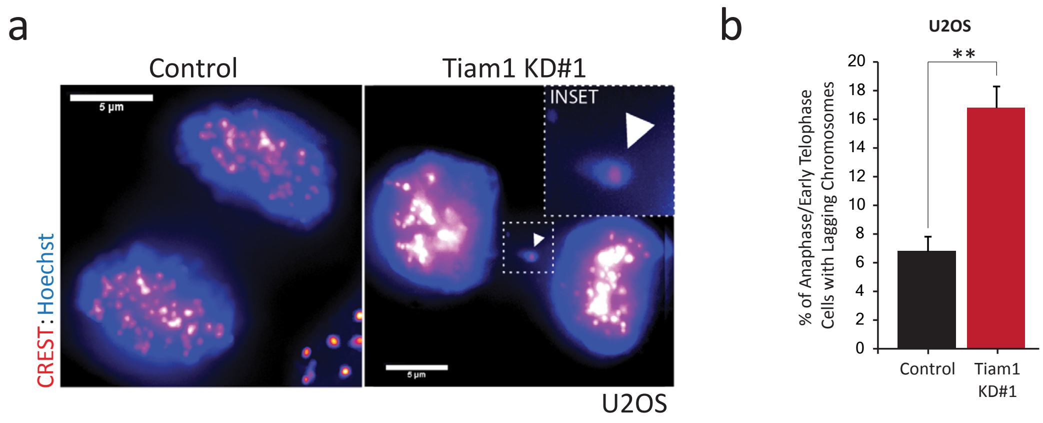

Figure S5

a) Images of U2OS cells (stained with Hoechst and CREST as a centromere marker, with a lagging chromosome at telophase in the cell treated with Tiam1 KD#1 siRNA (marked with an arrowhead). Scale bars are 5μm. b) Quantification of control and Tiam1 knockdown U2OS cells with lagging chromosomes at anaphase and early telophase from three independent experiments. ** $p<0.001$ (t-test); error bars show S.E.M.

Journal of Cell Science • Supplementary information
