# Supplementary material for: The RAC1 activator Tiam1 regulates centriole duplication through controlling PLK4 levels
Source: J Cell Sci. 2021 Apr 15;134(7):jcs252502. doi: 10.1242/jcs.252502 (PMC8075378; doi:10.1242/jcs.252502)
Supplement: Reviewer comments [file joces-134-252502_review_history.pdf]

Journal of Cell Science

The RAC1 activator Tiam1 regulates centriole duplication through controlling PLK4 levels

Andrew P. Porter, Hannah Reed, Gavin R. M. White, Erinn-Lee Ogg, Helen J. Whalley and Angeliki Malliri
DOI: 10.1242/jcs.252502

Editor: David Glover

Review timeline
Original submission: 31 July 2020
Editorial decision: 29 September 2020
First revision received: 13 January 2021
Editorial decision: 10 February 2021
Second revision received: 25 February 2021
Accepted: 1 March 2021

Original submission

<u>First decision letter</u>

MS ID#: JOCES/2020/252502

MS TITLE: The RAC1 activator Tiam1 regulates centriole duplication through controlling PLK4 levels

AUTHORS: Andrew Phillip Porter, Gavin R. M. White, Erinn-Lee Ogg, Helen J Whalley, and Angeliki Malliri
ARTICLE TYPE: Research Article

We have now reached a decision on the above manuscript.

To see the reviewers' reports and a copy of this decision letter, please go to: https://submit-jcs.biologists.org and click on the 'Manuscripts with Decisions' queue in the Author Area. (Corresponding author only has access to reviews.)

As you will see, the reviewers raise a number of substantial criticisms that prevent me from accepting the paper at this stage. They suggest, however, that a revised version might prove acceptable, if you can address their concerns. If you think that you can deal satisfactorily with the criticisms on revision, I would be pleased to see a revised manuscript. We would then return it to the reviewers.

We are aware that you may currently be unable to access the lab to undertake experimental revisions. If it would be helpful, we encourage you to contact us to discuss your revision in greater detail. Please send us a point-by-point response indicating where you are able to address concerns raised (either experimentally or by changes to the text) and where you will not be able to do so within the normal timeframe of a revision. We will then provide further guidance. Please also note that we are happy to extend revision timeframes as necessary.

Please ensure that you clearly highlight all changes made in the revised manuscript. Please avoid using 'Tracked changes' in Word files as these are lost in PDF conversion.

© 2021. Published by The Company of Biologists under the terms of the Creative Commons Attribution License (https://creativecommons.org/licenses/by/4.0/).

I should be grateful if you would also provide a point-by-point response detailing how you have dealt with the points raised by the reviewers in the 'Response to Reviewers' box. Please attend to all of the reviewers' comments. If you do not agree with any of their criticisms or suggestions please explain clearly why this is so.

Reviewer 1

Advance summary and potential significance to field

In this work, Porter and collaborators propose the involvement of Tiam1, a GEF of RAC1, on the regulation of Plk4 levels and therefore with a role on the centriole duplication pathway. Plk4, the master regulator of centriole duplication, is a kinase known to undergo a tight level regulation. Several publications demonstrated the relevance of the β-TRCP in controlling the levels of plk4, and it is well known that knocking down β-TRCP leads to centriole overduplication, in different organisms and cells tested. The authors propose that Tiam1, known to interact with β-TRCP, can control Plk4 levels without the requirement of Rac1 activation.
This is an exciting hypothesis, and the novelty of this work is the connection between Tiam1 and Centriole over-duplication. However, the authors lack to prove it.
The PCM marker pericentrin or the anti-centrin antibody cannot be used to "mark individual centrioles" (line 80).
There are commercially available and reliable antibodies that recognise essential centriolar proteins. This is the case of antibodies against CEP152, CPAP/SAS4, STIL or SAS6. These last two centriolar proteins are actually included in the scheme in Supplementary Figure S1a.
So far, the existence of centriole overduplication is still not evident and the paper needs some aditional clarifications as pointed below. For this reason, I cannot advise this work for publication. I consider the paper to be too preliminary and not suitable for publication in JCS as it is.

Comments for the author

Please find below some observations regarding some details in the paper.
Major Points
1. In Figure 1c, representative images for the Tiam KD#1 are missing, and insets from KD#2 and KD#3 do not match the original images. Regarding the pericentrin staining, it is not easy to visualise that there are extra centrioles in these images. In KD#3, there is an additional spot of centrin in the merged image that was neglected. The choices of dots to be quantified need to be better explained in the text. In agreement with previous comments, the Y-axis of the graphs in figure 1 (and other figures) do not correspond to abnormal centriole number (as indicated), but instead to the number of centrin dots/ cell.
2. Also, in supplementary figure 1b-d, in the experiments done with MCF7 cells, centriole over-duplication is reported, but the extra centrin 1 dots do not parallel any effect in the PCM protein pericentrin. Would not the authors expect to see more than two pericentrin dots in the case of centriole over-duplication?
3. After the ice-cold treatment, the levels of pericentrin seem to be higher in the control cells. Is there any explanation for this observation? After executing an ice-cold treatment, the staining for alpha-tubulin is a standard procedure and need to be included.
4. Centrosome amplification is usually associated with cell cycle delay or arrest and apoptosis. How do Porter and collaborators explain that Tiam 1 Knockdown does not affect the cell cycle progression or leads to an increase in apoptosis at least in U2OS cells that have a wild-type P53?
5. The Plk4 levels in Figure 2a are not representative of the average shown in the graph (figure 2b). Moreover, if centriole over-duplication occurs, one would expect more than two PLk4 dots/ centrosome as revealed in Figure 2 a and c.
6. Is the GEF mutant form of Tiam 1 able to localise to the centrosomes? Can this influence the ability of interaction of the β-TRCP with Plk4?
7. In figure 4d, there is a duplication of the same image- chromosomes spread for Tiam KD#1 and TiamKD #2 are the same. These images are shown with unmatched magnifications, and different numbers of chromosomes are indicated for the same karyotype. Scale bars should be re-analysed for Tiam 1 KD cells and control.

© 2021. Published by The Company of Biologists under the terms of the Creative Commons Attribution License (https://creativecommons.org/licenses/by/4.0/).

Minor points
1. Page 2, line 42-43, the authors wrote: “However, this gives rise to lagging chromosomes and ultimately, aneuploidy. The presence of extra centrosomes is not a direct cause of chromosome aneuploidy. It was shown that extra centrosomes increase the chance of merotelic kinetochore-MT attachments, that the spindle checkpoint cannot recognise, which can be the support for the existence of aneuploidy and chromosome instability in tumours with extra-centrosomes.
2. Page 3, line 47-48. Porter et al. wrote that “Indeed, centrosome amplification alone is sufficient for the initiation of tumorigenesis.” This statement is still controversial and not widely accepted.

Reviewer 2

Advance summary and potential significance to field

In this paper, the authors propose that Tiam1, a RAC1 GEF, can regulate Plk4 levels and hence centriole duplication. This is potentially an exciting finding, which if definitively proven could be highly significant. The role of β-TRCP in promoting Plk4 destruction is well characterized. Here the authors propose that the interaction of Tiam1 with β-TRCP, can control Plk4 levels without Rac1 activation. I have however, a single outstanding criticism of their findings – a failure to demonstrate that the effects they record are upon centrioles per se. The markers they use for immunostaining are insufficient to reach these conclusions. In the minimum, the experiments should be repeated using markers for the centriole cartwheel or ones that are known to associate with the elongating centriolar microtubules. At best, they should support their findings with EM observations.

Comments for the author

In this paper, the authors propose that Tiam1, a RAC1 GEF, can regulate Plk4 levels and hence centriole duplication. This is potentially an exciting finding, which if definitively proven could be highly significant. The role of β-TRCP in promoting Plk4 destruction is well characterized. Here the authors propose that the interaction of Tiam1 with β-TRCP, can control Plk4 levels without Rac1 activation. I have however, a single outstanding criticism of their findings – a failure to demonstrate that the effects they record are upon centrioles per se. The markers they use for immunostaining are insufficient to reach these conclusions. In the minimum, the experiments should be repeated using markers for the centriole cartwheel or ones that are known to associate with the elongating centriolar microtubules. At best, they should support their findings with EM observations.

Reviewer 3

Advance summary and potential significance to field

of centriole duplication and decrease or increase in PLK4 levels can lead to loss or amplification of centrosomes, respectively. Thus, in order to maintain accurate centrosome numbers, PLK4 levels and activity need to be controlled.

In this manuscript, Porter et al describe a novel mechanism important to regulate PLK4 and centriole numbers. They found that the Rac1 GEF Tiam1 plays a role in regulating centriole numbers by limiting PLK4 at the centrioles. Tiam1 depletion leads to increase centriole numbers in several cell lines and this is accompanied increased levels of PLK4 at the centrioles. This new Tiam1 function is independent of its GEF activity but relies on the N-terminal domain that contains beta-TRCP binding sites. Previously the authors found that Tiam1 via its GEF activity is important for centrosome separation in prophase, highlighting that Tiam1 could play different roles at the centrosomes. In addition, loss of Tiam1 also leads to chromosome missegregation, which could be due to centriole overduplication, and aneuploidy.

© 2021. Published by The Company of Biologists under the terms of the Creative Commons Attribution License (https://creativecommons.org/licenses/by/4.0/).

Comments for the author

Overall, the data presented here is novel, of high quality and will be of interest to cell biologists, therefore I would support its publication at Journal of Cell Science. However, there are several issues, highlighted below, that should be addressed by the authors to clarify and/or strengthen their conclusions.

#1. In the experiments where the authors quantify centriole amplification in interphase are these always assessed in S-phase cells? Since the control cells usually have already duplicated centrosomes I assume that is the case but that could be clearer and if PCNA staining is used that should also be mentioned. In supplementary figure 1c-d control MCF-7 cells only have one centrosome. Is this experiment quantified in a different way?

#2. In supplementary figure 1e, images before ice incubation should be provided to demonstrate for example the effect of loss of microtubules on centriolar satellites.

#3. The authors should show examples of images of centrioles associated with the quantification presented in supplementary figure 1l.

#4. Plk4 levels at the centrosome should be normalised to cell cycle phase. It is unclear if that was done in figure 2.

#5. The authors write on pages 5-6 that 20 nM of centrinone had very little or no effect on centriole number in control cells. However, this is not quantified. What is shown in figure 2g are cells with excess centrioles. Centrinone treatment leads to loss of centrioles, not excess.

Thus, the authors should clarify the text to highlight only what was assessed. If they have data for centriole number (loss of gain) than they should add it.

#6. The authors show in figure 3e that the Tiam1 C1199 and AA mutants do not bind beta-TRCP and thus they cannot regulate PLK4 levels and rescue centriole overduplication.
However, this is not completely shown. Do the authors know if the Tiam1 GEF mutant can still bind to beta-TRCP? Or does the expression of C1199 and AA mutants prevent increase PLK4 levels at the centrioles? This would help understand if the role of Tiam1 in centriole duplication is via beta-TRCP.

#7. It is unclear to me why centriole overduplication in Tiam1 depleted cells, which is around 25%, only generates very few cells with extra centrosomes in mitosis, as seen in supplementary figure 4b. Is it because these extra centrioles are immature? Or lost before mitosis? Did the authors tried to use different centriole markers, for example to stain distal appendages (mother centrioles) to see if these can mature?

#8. The authors found that despite low levels of centrosome amplification in mitosis, the levels of chromosome missegregation shown in figure 4b are around 20-25%. This suggests that perhaps Tiam1 loss leads to other mitotic defects that contribute the chromosome missegregation phenotype. It would help if the authors could show the % of chromosome missegregation in cells with normal and extra centrosomes. In addition, assessing chromosome missegregation in cells expressing Tiam1 mutants that rescue or not centriole overduplication would show if the segregation defects are only a result of centriole ovrduplication or not. At present, this is not clear.

First revision

<u>Author response to reviewers' comments</u>

We thank the reviewers for their careful reading of our manuscript and their constructive comments on how to strengthen the evidence we present. In response we have added a number of

© 2021. Published by The Company of Biologists under the terms of the Creative Commons Attribution License (https://creativecommons.org/licenses/by/4.0/).

new experiments as suggested, as well as edits to the text and existing figures, and below we outline how these address the points raised.

Response to Reviewer 1

Reviewer 1 Advance Summary and Potential Significance to Field:
In this work, Porter and collaborators propose the involvement of Tiam1, a GEF of RAC1, on the regulation of Plk4 levels and therefore with a role on the centriole duplication pathway. Plk4, the master regulator of centriole duplication, is a kinase known to undergo a tight level regulation. Several publications demonstrated the relevance of the β-TRCP in controlling the levels of plk4, and it is well known that knocking down β-TRCP leads to centriole overduplication, in different organisms and cells tested. The authors propose that Tiam1, known to interact with β-TRCP, can control Plk4 levels without the requirement of Rac1 activation.
This is an exciting hypothesis, and the novelty of this work is the connection between Tiam1 and Centriole over-duplication. However, the authors lack to prove it.
The PCM marker pericentrin or the anti-centrin antibody cannot be used to "mark individual centrioles" (line 80).
There are commercially available and reliable antibodies that recognise essential centriolar proteins. This is the case of antibodies against CEP152, CPAP/SAS4, STIL or SAS6. These last two centriolar proteins are actually included in the scheme in Supplementary Figure S1a.
So far, the existence of centriole overduplication is still not evident and the paper needs some aditional clarifications as pointed below. For this reason, I cannot advise this work for publication. I consider the paper to be too preliminary and not suitable for publication in JCS as it is.

We are pleased that the Reviewer considers this to be an exciting and novel hypothesis, and we appreciate their clear summary of our findings. We understand their criticism that *'pericentrin or the anti-centrin antibody cannot be used to "mark individual centrioles"'*, and have therefore performed a series of experiments imaging additional centriole and centrosome markers – namely CP110, SAS6 and Gamma-Tubulin. We chose CP110 and SAS6 because they mark distinct parts of the centriole - SAS6 is part of the 'cartwheel' at the base of the procentriole (mitotic centrosomes normally contain one puncta of SAS6), while CP110 caps the distal end of centrioles in non-ciliated cells (mitotic centrosomes normally contain two puncta of CP110).

For this experiment, we specifically imaged mitotic cells, to ensure we were comparing a population of cells at the same stage of the cell cycle, all of which had been through at least one round of centriole duplication in S-phase. Consistent with our previous findings using Centrin staining alone, we see that Tiam1 knockdown leads to an increase in cells with excess puncta of both CP110 and SAS6 associated with the centrosome (as marked by Pericentrin or Gamma-Tubulin). This data has been included in the paper as part the new Figure 2 in the revised manuscript, and the data is shown here:

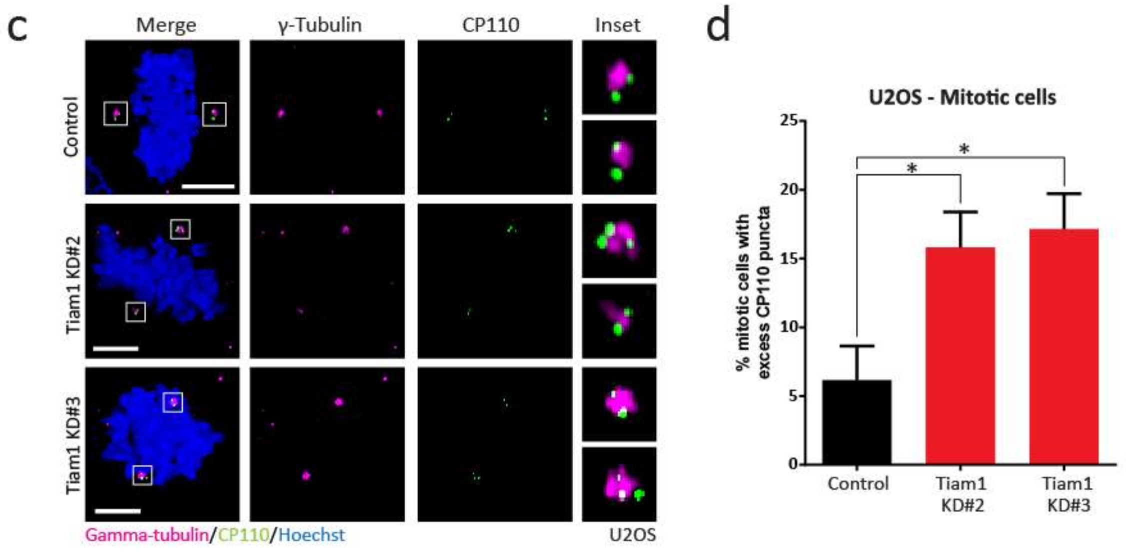

© 2021. Published by The Company of Biologists under the terms of the Creative Commons Attribution License (https://creativecommons.org/licenses/by/4.0/).

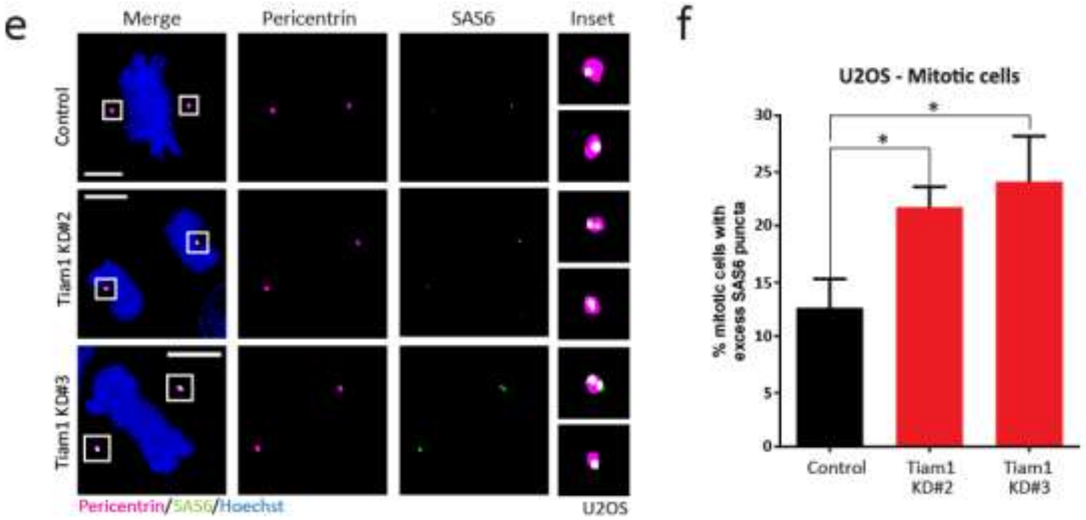

We have also changed the description of our original Centrin-staining experiments in the main text, following the Reviewer's direction, so that it now reads:

"). To test this, we depleted Tiam1 from U2OS cells using RNAi (Figure 1b). An asynchronous population of control and knockdown cells were stained with antibodies against Pericentrin (to mark the pericentriolar material surrounding the centrioles (Doxsey et al., 1994)) and Centrin (an integral structural component of centrioles themselves (Bornens, 2002)) (Figure 1c). Depletion of Tiam1 led to an increase in the percentage of cells displaying excess Centrin puncta (i.e. greater than 2 Centrin puncta per centrosome) (Figure 1c). Using three independent siRNA sequences targeting Tiam1, we saw a significant increase in the number of cells displaying these additional Centrin puncta compared with control treated cells (Figure 1d)."

The increase in the percentage of cells containing excess puncta of both these markers is very similar to that seen with Centrin staining alone. We feel this strongly supports our overall conclusion that depletion of Tiam1 is producing a true defect in centriole duplication, although we continue to be careful throughout the paper to ensure there is an appropriate separation between description of results - i.e. counting of excess puncta - and the ultimate conclusion that these represent an increase in centriole number.

Following the description of our findings in the main text, we have therefore added this summary statement:

"Given the increase in active, mitotic centrosomes containing excess puncta of three centriole markers marking distinct components of the centriole, and that the Centrin puncta are stable after cold treatment, we propose that Tiam1 depletion leads to centriole overduplication."

Further details on other experiments which support this are presented in response to other points below.

Reviewer 1 Comments for the Author:

Please find below some observations regarding some details in the paper.
<u>Major Points</u>
1. In Figure 1c, representative images for the Tiam KD#1 are missing, and insets from KD#2 and KD#3 do not match the original images.

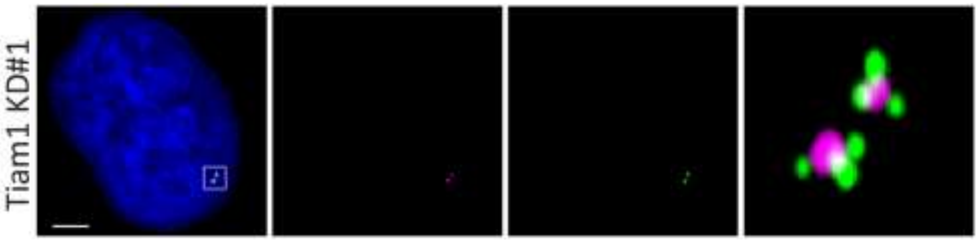

© 2021. Published by The Company of Biologists under the terms of the Creative Commons Attribution License (https://creativecommons.org/licenses/by/4.0/).

We have added the missing Tiam1 KD#1 figure panel, which is shown here.
The insets from KD#2 and KD#3 are cropped versions of the Pericentrin and Centrin channels merged, but without the Hoechst staining. We have added this as a note to the legend for Figure 1, which reads:

“Panels labelled ‘Inset’ show a magnified version of the region marked with a white box in the merge, with Hoechst omitted for clarity; a similar principle applies to Figures 1f and 1i.”

Regarding the pericentrin staining, it is not easy to visualise that there are extra centrioles in these images. In KD#3, there is an additional spot of centrin in the merged image that was neglected. The choices of dots to be quantified need to be better explained in the text.

We have added extra detail to our methods section to explain that we only count spots of Centrin or other centriole markers if they are associated with a marker of pericentriolar material:

“Puncta were only counted if they were associated with Pericentrin or Gamma-Tubulin staining as markers of pericentriolar material.”

In agreement with previous comments, the Y-axis of the graphs in figure 1 (and other figures) do not correspond to abnormal centriole number (as indicated), but instead to the number of centrin dots/ cell.

We agree with the point the Reviewer makes that Centrin staining alone is insufficient to truly identify an increase in centriole duplication, and therefore the Y-axis labels should be adjusted. While we feel that the combined weight of evidence across the paper supports this conclusion (especially with the addition of data on the staining of SAS6 and CP110), we have adjusted the Y-axis legends on the graphs throughout the manuscript to unambiguously describe the specific findings of each experiment – i.e. the observation of cells within increased puncta of Centrin. We also apply this approach to the newly-added data on SAS6 and CP110 puncta. An example of this change is shown here:

Original Y-axis legend

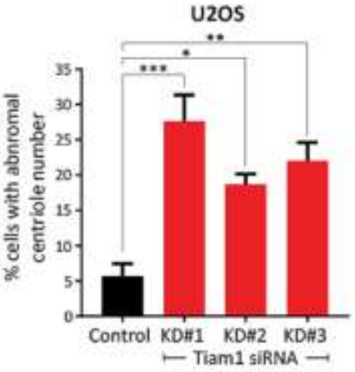

Modified Y-axis legend

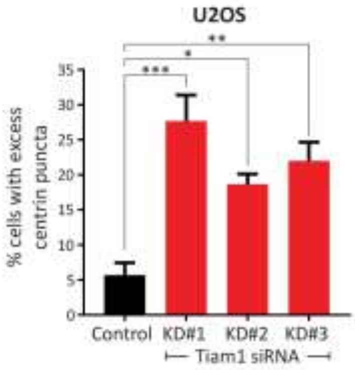

We have made similar changes to the main text and figure legends throughout the manuscript.

2. Also, in supplementary figure 1b-d, in the experiments done with MCF7 cells, centriole over-duplication is reported, but the extra centrin 1 dots do not parallel any effect in the PCM protein pericentrin. Would not the authors expect to see more than two pericentrin dots in the case of centriole over-duplication?

We have taken the decision to remove the data relating to MCF7 cells from the paper (original Supplementary Figure 1b-d). While these data do show changes in centrin staining in a third cell type, we only use the cell line in this one experiment with a single siRNA oligo, and so have removed the panels.

3. After the ice-cold treatment, the levels of pericentrin seem to be higher in the control cells. Is

© 2021. Published by The Company of Biologists under the terms of the Creative Commons Attribution License (https://creativecommons.org/licenses/by/4.0/).

there any explanation for this observation? After executing an ice-cold treatment, the staining for alpha-tubulin is a standard procedure and need to be included.

We believe the high Pericentrin staining seen in Supplementary Figure 2b (new) is due to natural variance between cells; we did not record any particular trend towards changes in Pericentrin staining in these experiments.

The third panel depicts αTubulin staining, showing hazy, depolymerised cytoplasmic staining (these images are now part of Supplementary Figure 2). Unfortunately we do not have matched images for all the experiments pre- and post-cold treatment, but we include for the reviewer an example cell which had been returned to 37ºC to demonstrate that the microscopy method can detect microtubules, strongly indicating that after 30 minutes on ice the cells have completely depolymerised their microtubules.

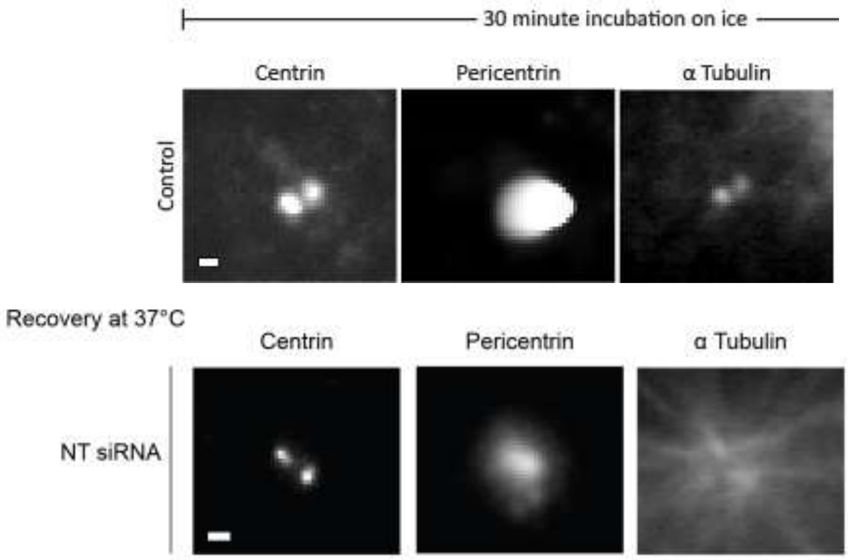

4. Centrosome amplification is usually associated with cell cycle delay or arrest and apoptosis. How do Porter and collaborators explain that Tiam 1 Knockdown does not affect the cell cycle progression or leads to an increase in apoptosis at least in U2OS cells that have a wild-type P53?

As the reviewer suggests, we expected to see a change in cell cycle progression, given our other findings. The reason for the seemingly normal FACS data remains an open question, and following the reviewer's point we now address this more in the discussion. We have also not explicitly tested for a change in apoptosis, which may be occurring following Tiam1 knockdown, and again we refer to this in our discussion, part of which now reads:

"As both the U2OS and HCT116 cells used here have a functioning p53 pathway, it is possible that the centriole defects are triggering an apoptotic response such that cells with excess centrosomes are being lost from the population, or that a more subtle change in cell cycle progression is occurring that we have yet to detect."

5. The Plk4 levels in Figure 2a are not representative of the average shown in the graph (figure 2b).

We have replaced the images in original Figure 2a (now Figure 3a in the revised manuscript) with cells where the PLK4 levels are closer to the average values, and these panels are shown here:

© 2021. Published by The Company of Biologists under the terms of the Creative Commons Attribution License (https://creativecommons.org/licenses/by/4.0/).

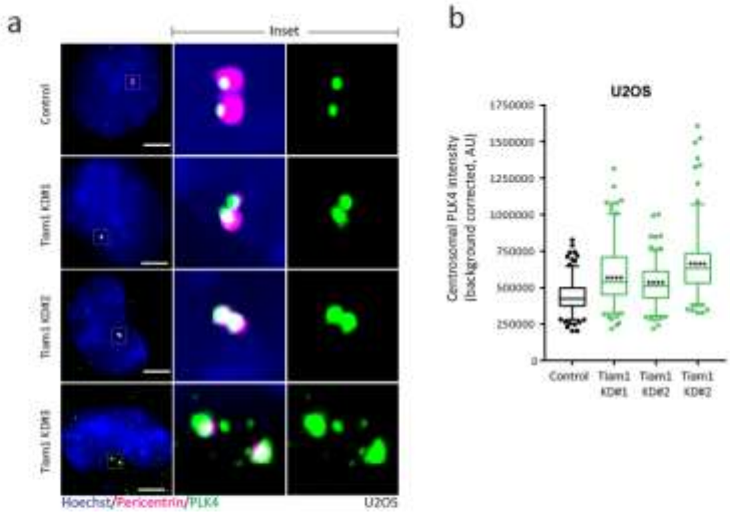

Moreover, if centriole over-duplication occurs, one would expect more than two PLk4 dots/ centrosome as revealed in Figure 2 a and c.

We agree with the Reviewer that there may be more an increase in the PLK4 puncta given the changes in centriole duplication. However, both this experiment and the newly-added PLK4 imaging (now incorporated into the new Figure 4) were performed to maximise sensitivity over resolution, and we would not feel confident drawing conclusions on the precise number of PLK4 puncta in these cells. However, an increase in the number of puncta and an increase in total PLK4 should both register as an increase in centrosomal PLK4 intensity, which is what we observe in these experiments.

Further imaging – at higher resolution – would be required to determine whether there are more subtle changes in PLK4 puncta and/or localisation, and we have added this point to the discussion, where we now say:

"Our PLK4 imaging setups using both widefield and confocal microscopy were optimised for intensity measurements; more specialist super-resolution imaging (such as in (Ohta et al., 2018)) may determine whether there are subtler changes to PLK4 localisation or the number of PLK4 puncta."

6. Is the GEF mutant form of Tiam 1 able to localise to the centrosomes? Can this influence the ability of interaction of the ß-TRCP with Plk4?

We show localisation of the GEF mutant form of Tiam1 to centrosomes in Supplementary Figure 4a, and have added a clarifying note to the text, where we say:

"First we confirmed that GEF* Tiam1 localised to centrosomes similarly to wild-type Tiam1 (Supplementary Figure 4a)."

We have performed a new co-immunoprecipitation experiment which demonstrates that the GEF mutant of Tiam1 is able to bind to ß-TRCP. This blot has been added to the paper as Supplementary Figure 4g and is also shown here:

© 2021. Published by The Company of Biologists under the terms of the Creative Commons Attribution License (https://creativecommons.org/licenses/by/4.0/).

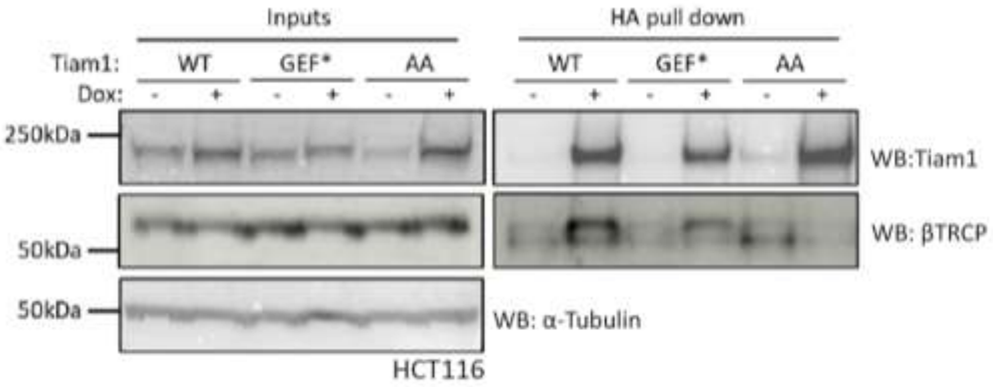

Along with our rescue experiments in Figure 4 and Supplementary Figure 4, these suggest that the GEF mutant of Tiam1 functions like wild-type Tiam1 in relation to ß-TRCP binding and PLK4 modulation.

7. In figure 4d, there is a duplication of the same image- chromosomes spread for Tiam KD#1 and TiamKD #2 are the same. These images are shown with unmatched magnifications, and different numbers of chromosomes are indicated for the same karyotype. Scale bars should be re-analysed for Tiam 1 KD cells and control.

We thank the reviewer for spotting this duplication, which arose during production of the figure. We apologise for the error, and have corrected the figure. To ensure consistency in scale and presentation, we returned to the raw microscopy images and have remade the figure, which now shows show correct images for Control, Tiam1 KD#1 and Tiam1 KD#2. The corrected panel, now found in Figure 5d, is shown here:

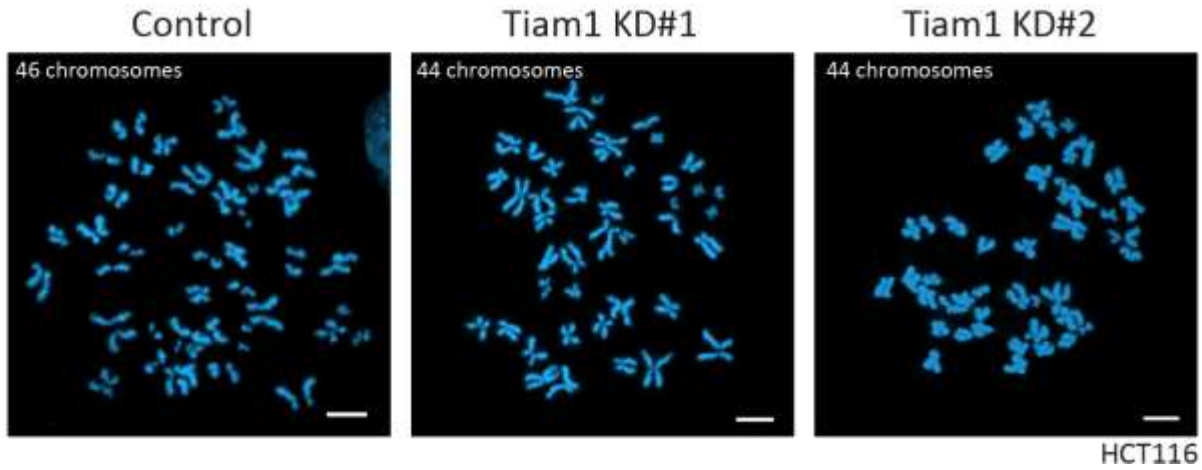

Minor points
1. Page 2, line 42-43, the authors wrote: "However, this gives rise to lagging chromosomes and ultimately, aneuploidy. The presence of extra centrosomes is not a direct cause of chromosome aneuploidy. It was shown that extra centrosomes increase the chance of merotelic kinetochore-MT attachments, that the spindle checkpoint cannot recognise, which can be the support for the existence of aneuploidy and chromosome instability in tumours with extra-centrosomes.

We thank the Reviewer for this useful clarification, and have adjusted the text of the introduction so that it now reads:

"Divisions proceed in such cells through clustering of extra centrosomes to form pseudo-bipolar spindles. However, the formation of multipolar intermediates increases the incidence of merotelic microtubule-kinetochore attachments to chromosomes which do not trigger the

© 2021. Published by The Company of Biologists under the terms of the Creative Commons Attribution License (https://creativecommons.org/licenses/by/4.0/).

spindle attachment checkpoint. These in turn can give rise to lagging chromosomes and ultimately aneuploidy (Ganem et al., 2009).”

2. Page 3, line 47-48. Porter et al. wrote that “Indeed, centrosome amplification alone is sufficient for the initiation of tumorigenesis.” This statement is still controversial and not widely accepted.

We appreciate the Reviewers insight into this statement, and have modified our summary sentence to better reflect the controversial nature of this point, so that the introduction text now reads:

“Indeed, it has been reported that centrosome amplification alone is sufficient for the initiation of tumourigenesis (Levine et al., 2017).“

Response to Reviewer 2

Reviewer 2 Advance Summary and Potential Significance to Field & Comments for the Author:
In this paper, the authors propose that Tiam1, a RAC1 GEF, can regulate Plk4 levels and hence centriole duplication. This is potentially an exciting finding, which if definitively proven could be highly significant. The role of β-TRCP in promoting Plk4 destruction is well characterized. Here the authors propose that the interaction of Tiam1 with β-TRCP, can control Plk4 levels without Rac1 activation.
I have however, a single outstanding criticism of their findings – a failure to demonstrate that the effects they record are upon centrioles per se. The markers they use for immunostaining are insufficient to reach these conclusions.
In the minimum, the experiments should be repeated using markers for the centriole cartwheel or ones that are known to associate with the elongating centriolar microtubules. At best, they should support their findings with EM observations.

We appreciate the Reviewers assessment that this work is exciting and potentially highly significant. However, we understand their criticism that the markers we have used – i.e. Centrin and Pericentrin – are insufficient to fully support our claim that the extra puncta we see are true centrioles.

To address this, we have performed experiments using additional centriole and centrosome markers, namely SAS6 (which marks the centriole ‘cartwheel’ at the base of the developing procentriole), CP110 (which caps the distal ends of centrioles) and gamma-Tubulin (as an additional marker of pericentriolar material). We now show that depletion of Tiam1 with two distinct siRNAs leads to a significant increase in the number of cells with excess puncta of both SAS6 and CP110. This increase is consistent with the increase we see for Centrin, and so these additional centriolar markers strongly support our conclusions that the defects we are seeing are in centrioles.

Further, these new experiments specifically examined changes specifically in mitotic cells, which have therefore been through at least one round of S-phase (where centriole duplication occurs). We have therefore observed excess puncta of three distinct centriolar markers - Centrin, SAS6 and CP110 - in centrosomes engaged in the formation of the mitotic spindle, making it highly likely that these excess puncta do indeed represent bona fide centrioles. This data has been added to the newly-created Figure 2, (alongside a modified version of the mitotic Centrin staining figure which was previously in Supplementary Figure 1, as this fits well with our new findings). We present the CP110 and SAS6 panels here:

© 2021. Published by The Company of Biologists under the terms of the Creative Commons Attribution License (https://creativecommons.org/licenses/by/4.0/).

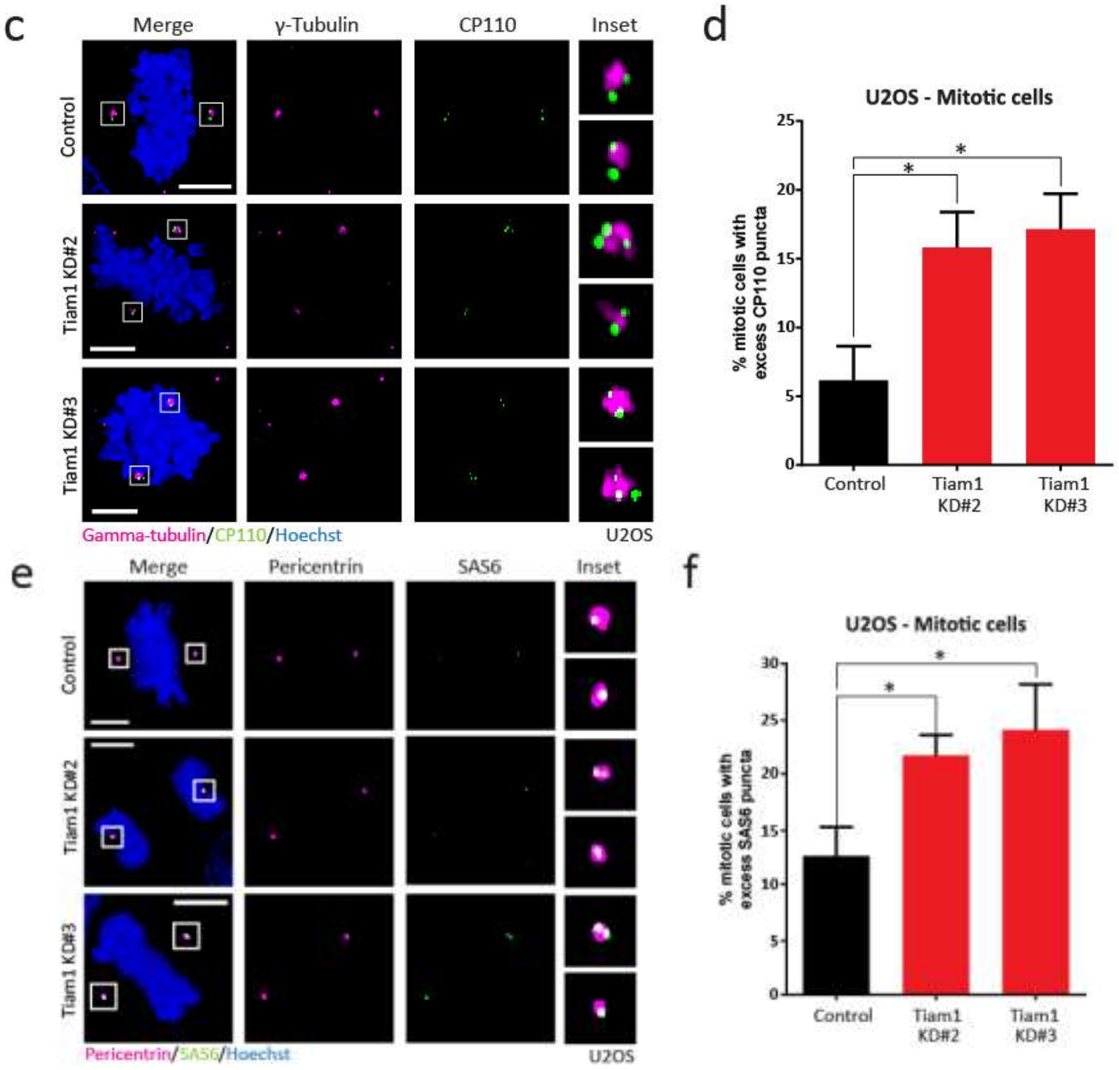

We have also adjusted the presentation of the data throughout the paper to reflect the Reviewers concerns, with an example shown here.

Original Y-axis legend

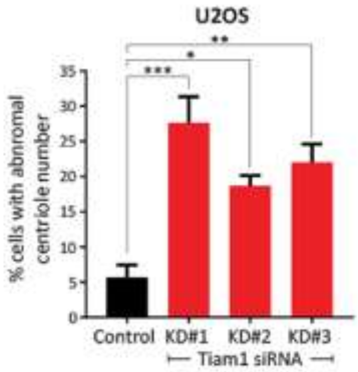

Modified Y-axis legend

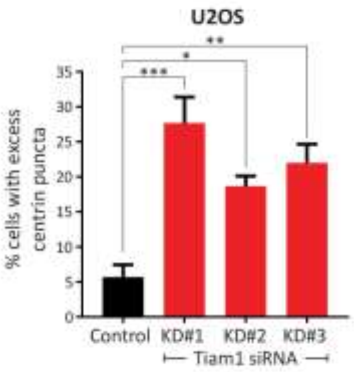

All figures reporting on changes in Centrin, SAS6 and CP110 now report the direct observation of the experiment – i.e. an increase in cells with excess puncta – rather than the more general claim that these are excess centrioles per se, which we understand is more than each figure by itself can show. We only draw this conclusion – that Tiam1 depletion affects centriole duplication - in the main text, where we feel that – on balance – it represents the most likely interpretation of the assembled data.

© 2021. Published by The Company of Biologists under the terms of the Creative Commons Attribution License (https://creativecommons.org/licenses/by/4.0/).

Response to Reviewer 3

Reviewer 3 Advance Summary and Potential Significance to Field:

In this manuscript, Porter et al describe a novel mechanism important to regulate PLK4 and centriole numbers. They found that the Rac1 GEF Tiam1 plays a role in regulating centriole numbers by limiting PLK4 at the centrioles. Tiam1 depletion leads to increase centriole numbers in several cell lines and this is accompanied increased levels of PLK4 at the centrioles. This new Tiam1 function is independent of its GEF activity but relies on the N-terminal domain that contains beta-TRCP binding sites. Previously the authors found that Tiam1 via its GEF activity is important for centrosome separation in prophase, highlighting that Tiam1 could play different roles at the centrosomes. In addition, loss of Tiam1 also leads to chromosome missegregation, which could be due to centriole overduplication, and aneuploidy.

We thank the reviewer for this clear summary of our findings.

Reviewer 3 Comments for the Author:
Overall, the data presented here is novel, of high quality and will be of interest to cell biologists, therefore I would support its publication at Journal of Cell Science. However, there are several issues, highlighted below, that should be addressed by the authors to clarify and/or strengthen their conclusions.

We are pleased that the Reviewer supports publication of our findings in Journal of Cell Science, and we are pleased to address their specific points below.

#1. In the experiments where the authors quantify centriole amplification in interphase are these always assessed in S-phase cells? Since the control cells usually have already duplicated centrosomes I assume that is the case but that could be clearer and if PCNA staining is used that should also be mentioned.

In our original submission, all the experiments quantifying centriole amplification analyse a mixed cell population (except for the original Supplementary Figure 1h and 1i which looked specifically at mitotic cells). The quantification therefore included cells in interphase and in mitosis (although in these asynchronous populations only a small percentage of cells were mitotic). Images were chosen from cells which are either in S-phase or G2 (based on centrosome status) for clarity of comparison, but no formal PCNA staining was used at that stage.
We appreciate the Reviewers point about clarity and we have therefore expanded our methods section to better describe our approach. We now say that:

"Unless otherwise stated, cells analysed are from an asynchronous population which includes interphase and mitotic cells."

A limitation of our original analysis is that it also includes cells in G1, which would only register as abnormal if their single centrosome contained more than three puncta of centrin, which they would have to have inherited from the abnormal parental cell. However, our new analysis of centriole overduplication using both SAS6 and CP110 was performed exclusively in a mitotic population, i.e. cells which have been through at least one round of S-phase. These changes, and those in the original Supplementary Figure 1h and 1i, are consistent with our bulk population measurement.

As described below, we have also used PCNA as an S-phase marker during additional PLK4 imaging and quantification. These additions, while in line with our original findings, strengthen our conclusions through more precise cell-cycle stage specific analysis.

In supplementary figure 1c-d control MCF-7 cells only have one centrosome. Is this experiment quantified in a different way?

These images were quantified in the same way as the other experiments in the paper - cells with one centrosome were counted as abnormal if they displayed greater than three Centrin puncta. However, we have decided to remove the data relating to MCF7 cells (original Supplementary Figure 1b-d), due to the relatively limited analysis performed in this cell line.

© 2021. Published by The Company of Biologists under the terms of the Creative Commons Attribution License (https://creativecommons.org/licenses/by/4.0/).

#2. In supplementary figure 1e, images before ice incubation should be provided to demonstrate for example the effect of loss of microtubules on centriolar satellites.

We agree with the reviewer that the images before ice incubation would be helpful in showing the loss of microtubules following cold treatment. Unfortunately when these experiments were conducted those images were not acquired, and quantification of centriole number was performed directly on the microscope, rather than through acquisition of images – except for several representative images of each condition. The α-Tubulin staining in the third panels does show a hazy cytoplasmic staining, and these are maximum z-projections, demonstrating a loss of cytoplasmic microtubules. An image showing microtubule re-nucleation after a return to 37oC was captured, and we include that here for the Reviewer to demonstrate that the system was capable of detecting cytoplasmic microtubules, emphasising their loss under cold treatment.

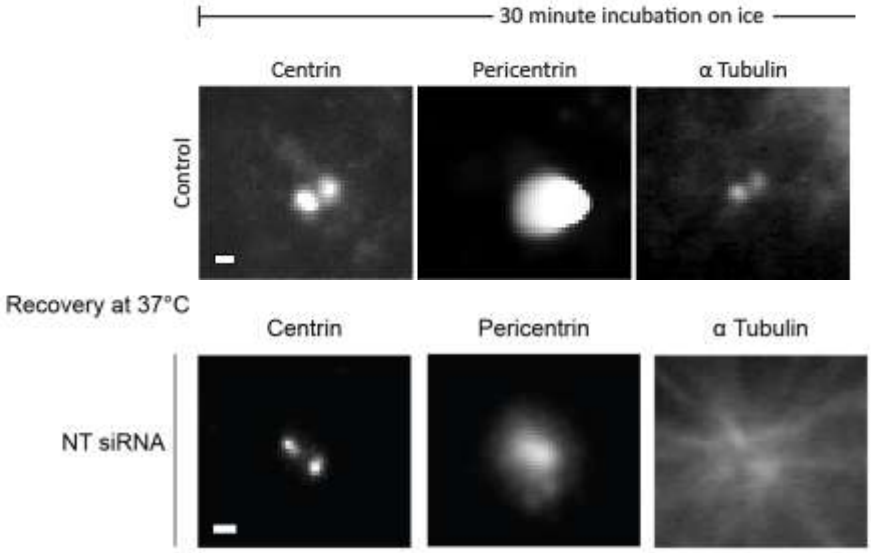

#3. The authors should show examples of images of centrioles associated with the quantification presented in supplementary figure 1l.

We have added these images now (to the new Supplementary Figure 2), and also show the panel here:

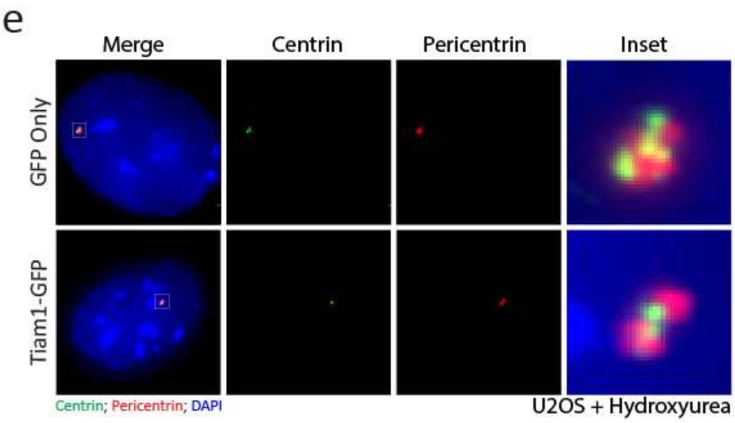

#4. Plk4 levels at the centrosome should be normalised to cell cycle phase. It is unclear if that was done in figure 2.

© 2021. Published by The Company of Biologists under the terms of the Creative Commons Attribution License (https://creativecommons.org/licenses/by/4.0/).

As outlined in response to Point #1, our original analysis of PLK4 levels was performed on a mixed population of interphase cells (excluding any mitotic cells). We have clarified this point in the methods, where we say:

"Images were acquired from an asynchronous culture; intensity measurements were only performed on interphase cells."

We have subsequently performed new experiments imaging PLK4, using a point scanning confocal (Lecia SP8) with Hybrid Detectors in a quantitative photon-counting mode, and coupled this with PCNA staining for the detection of cells in S-phase. Analysing the intensity of centrosomal PLK4 staining in a purely S-phase population, we see again that depletion of Tiam1 leads to an increase in centrosomal PLK4, consistent with the imaging performed using a widefield microscope on a mixed interphase population. These images and analysis are now part of the new Figure 4 and Supplementary Figure 4, and we present part of this data here (a fuller version is shown in response to point #6 below).

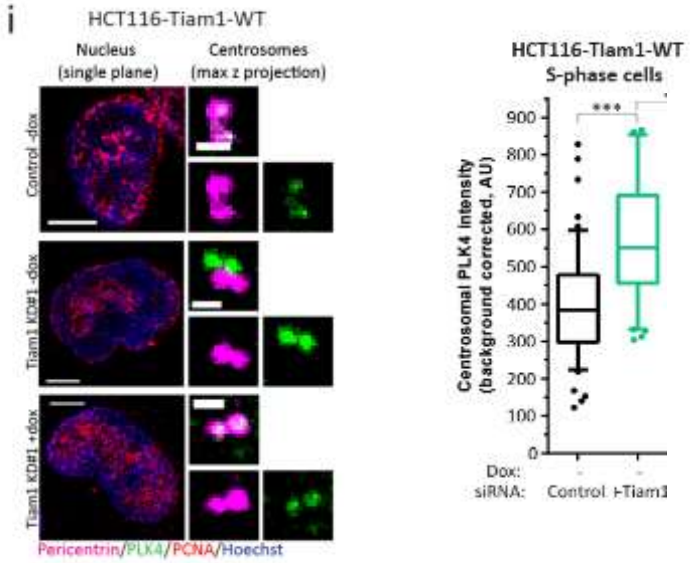

#5. The authors write on pages 5-6 that 20 nM of centrinone had very little or no effect on centriole number in control cells. However, this is not quantified. What is shown in figure 2g are cells with excess centrioles. Centrinone treatment leads to loss of centrioles, not excess. Thus, the authors should clarify the text to highlight only what was assessed. If they have data for centriole number (loss of gain) than they should add it.

We appreciate the Reviewer's direction that this experiment should be written more clearly, and have re-written the main text to better describe what was done. We now say:

"We therefore treated HCT116 cells with the specific PLK4 inhibitor Centrinone (Wong et al., 2015) at 20 nM. Our preliminary experiments indicated that this concentration had a minimal effect on basal centriole duplication, with most control siRNA treated cells containing centrosomes with two Centrin puncta (data not shown). Tiam1 knockdown in the absence of the inhibitor led to an increase in cells with excess Centrin puncta as previously observed (Figure 3g). However, knockdown cells treated with Centrinone did not display an increase in cells with excess Centrin puncta compared to control-siRNA treated cells (Figure 3g). These findings demonstrate that the production of extra centrioles in Tiam1-depleted cells requires increased PLK4 activity."

In the methods we now state the details more explicitly:

"Centrinone Treatment
Following siRNA transfection and plating, cells were treated with 20nM Centrinone (Tocris Bioscience) or vehicle control for 3 days before fixation. In preliminary experiments, higher

© 2021. Published by The Company of Biologists under the terms of the Creative Commons Attribution License (https://creativecommons.org/licenses/by/4.0/).

concentrations of Centrinone led to an obvious decrease in cells with centrosomes (marked with Pericentrin) and increase in centrosomes with no Centrin puncta, However, 20nM Centrinone had relatively little effect on basal Centrosome and Centriole number (data not shown). For quantification, cells were marked as abnormal if they contained centrosomes with greater than 2 Centrin puncta, as in other experiments in the paper."

We have also changed the y-axis label on the chart to more accurately reflect what was being counted:

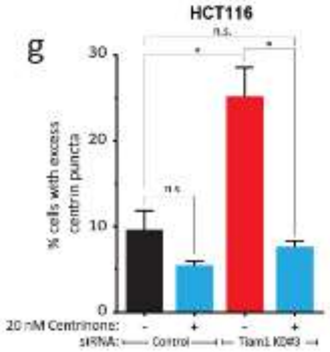

#6. The authors show in figure 3e that the Tiam1 C1199 and AA mutants do not bind beta-TRCP and thus they cannot regulate PLK4 levels and rescue centriole overduplication. However, this is not completely shown. Do the authors know if the Tiam1 GEF mutant can still bind to beta-TRCP?

We have now tested whether the GEF mutant of Tiam1 can bind βTRCP by performing a new coIP experiment. This demonstrates that the GEF mutant does indeed bind βTRCP, supporting our other evidence – from rescue experiments and suppression of hydroxyurea-induced centriole overduplication – that the GEF mutant performs the same as WT Tiam1 with respect to centriole duplication. The blot has been incorporated into Supplementary Figure 4, and is also shown here:

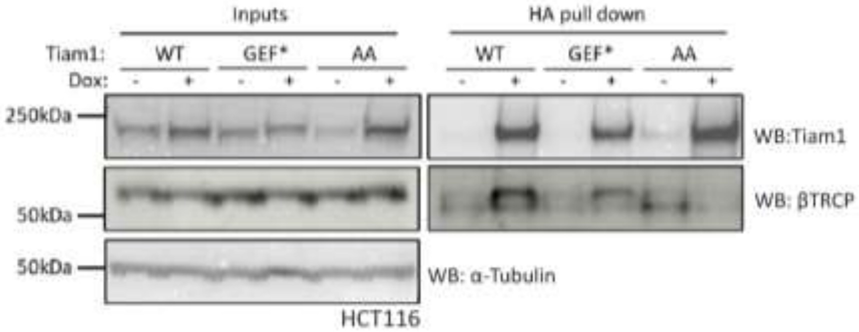

Or does the expression of C1199 and AA mutants prevent increase PLK4 levels at the centrioles? This would help understand if the role of Tiam1 in centriole duplication is via beta-TRCP.

We have performed a new set of rescue experiments looking specifically at PLK4 at the centrosome in cells in S-phase to address this point. We used the PCNA marker to distinguish S-phase cells, and imaged PLK4 using the Leica SP8 confocal microscope, with a hybrid detector in the quantitative photon counting mode. This provided a second imaging system, distinct from the widefield imaging we had already performed, and focused specifically on one phase of the cell cycle (whereas the other imaging was performed on a mixed population of interphase cells).
In support of our previous findings, we again saw a significant increase in centrosomal PLK4 after Tiam1 knockdown. We then performed a rescue experiment by re-expressing wild-type Tiam1 in a doxycycline-inducible manner, which significantly decreased the centrosomal PLK4 signal. This is shown in Figure 4 (with a second experiment shown in Supplementary Figure 4; disparities in the absolute measurements made it difficult to combine the data from the two experiments, so they are shown separately.) We also include the data here:

© 2021. Published by The Company of Biologists under the terms of the Creative Commons Attribution License (https://creativecommons.org/licenses/by/4.0/).

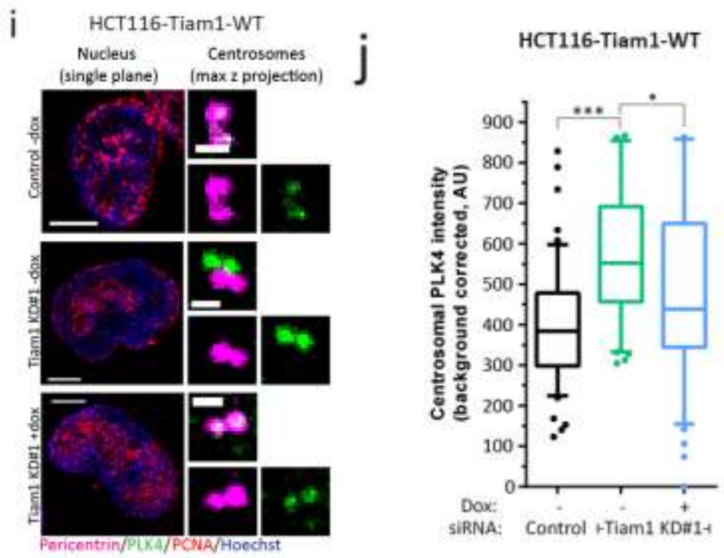

We then repeated the experiment, this time expressing the non-ß-TRCP binding Tiam1-AA mutant. While Tiam1 knockdown in these cells again increased centrosomal PLK4 levels in both experiments, we did not see any decrease in centrosomal PLK4 when expressing the mutant Tiam1-AA protein. The data is included in Figure 4 (with an additional experiment added to Supplementary Figure 4), and is also shown here:

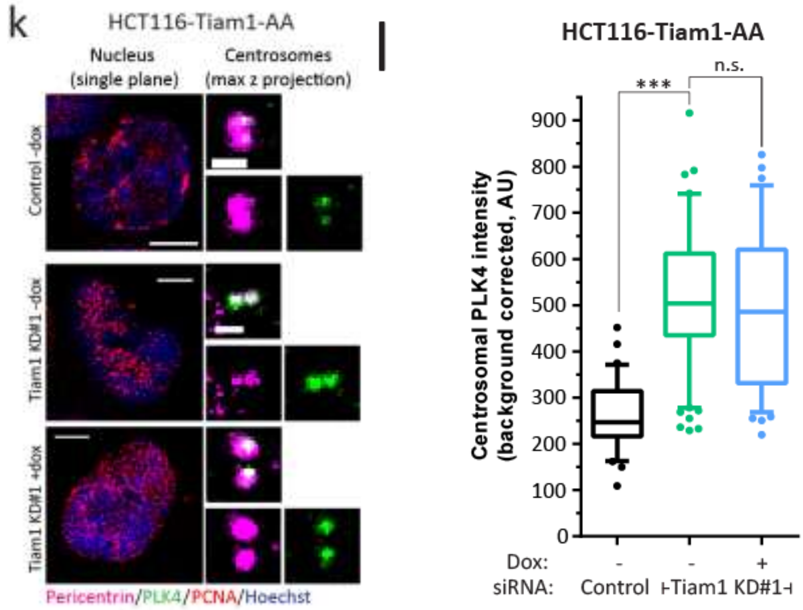

This new data strengthens our conclusion that Tiam1 requires the ability to bind to ß-TRCP to specifically regulate centrosomal PLK4 in S-phase.

#7. It is unclear to me why centriole overduplication in Tiam1 depleted cells, which is around 25%, only generates very few cells with extra centrosomes in mitosis, as seen in supplementary figure 4b. Is it because these extra centrioles are immature? Or lost before mitosis? Did the authors tried to use different centriole markers, for example to stain distal appendages (mother centrioles) to see if these can mature?

The reviewer highlights something that remains an open question at this point, and which we now address directly in our discussion. Seeing an increase in CP110 puncta in mitotic centrosomes following Tiam1 knockdown suggests that at least an early part of the centriole maturation process has occurred. We have some preliminary data which suggests that some of the extra structures contain CEP164, a component of the distal appendages, and an example image is shown here for

© 2021. Published by The Company of Biologists under the terms of the Creative Commons Attribution License (https://creativecommons.org/licenses/by/4.0/).

the reviewer, although these data are only from a pilot experiment so we have not included them in the final manuscript except for a reference in the discussion.

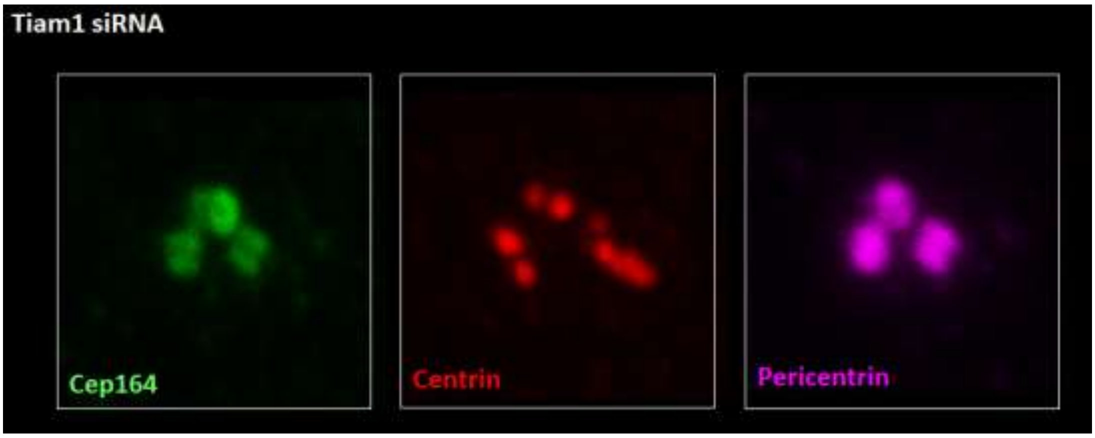

It is also possible that there is an increase in apoptosis and/or a more subtle change in cell cycle progression that we have not picked up with our current FACS analysis, and we have added these points to the discussion, where we now say:

"We show that loss of Tiam1 leads to an increase in lagging chromosomes at anaphase, with around 25% of knockdown cells presenting with lagging chromosomes. We also see centriole abnormalities in around 25% of the population, and analysis in mitotic cells shows that this often affects one pole of the mitotic spindle, generating asymmetric centriole numbers, which are a potent driver of chromosome missegregation (Cosenza et al., 2017). With only a small percentage of cells displaying full centrosome amplification, further analysis (such as staining with markers of more mature centrioles and electron microscopy reconstructions of the centriole structures) will be required to better understand this relatively low conversion rate, although the increase in CP110 staining suggests that these centriolar structures are undergoing at least part of the centriolar maturation process. As both the U2OS and HCT116 cells used here have a functioning p53 pathway, it is possible that the centriole defects are triggering an apoptotic response such that cells with excess centrosomes are being lost from the population, or that a more subtle change in cell cycle progression is occurring that we have yet to detect."

#8. The authors found that despite low levels of centrosome amplification in mitosis, the levels of chromosome missegregation shown in figure 4b are around 20-25%. This suggests that perhaps Tiam1 loss leads to other mitotic defects that contribute the chromosome missegregation phenotype. It would help if the authors could show the % of chromosome missegregation in cells with normal and extra centrosomes. In addition, assessing chromosome missegregation in cells expressing Tiam1 mutants that rescue or not centriole overduplication would show if the segregation defects are only a result of centriole ovrduplication or not. At present, this is not clear.

We agree that there may be other defects contributing to chromosome missegregation, and we reference our previous work showing misalignment of chromosomes during metaphase following Tiam1 depletion as one possible aspect. Regarding a rescue experiment, due to time constraints we have instead focused on rescuing the core PLK4 phenotype (see responses above).

While our analysis of lagging chromosomes did not directly measure centriole and centrosome number in the same data set, our SAS6 and CP110 mitotic cell analysis shows a high level of centriole asymmetry at the mitotic poles, which has been shown to give rise to a high level of lagging chromosomes. We have added more details on this to the main text where we now say:

"Chromosome mis-segregation can also occur through an imbalance of centriole numbers at the two poles of the mitotic spindle which leads to differences in microtubule nucleation between the poles and does not trigger the cellular error correction machinery (Cosenza et al., 2017).

© 2021. Published by The Company of Biologists under the terms of the Creative Commons Attribution License (https://creativecommons.org/licenses/by/4.0/).

Interestingly, detailed analysis of mitotic U2OS cells stained with CP110 and SAS6 revealed that all the cells with excess CP110 or SAS6 puncta had asymmetric centriole number at the poles, suggesting that this might strongly affect chromosome segregation (data not shown).”

Second decision letter

MS ID#: JOCES/2020/252502

MS TITLE: The RAC1 activator Tiam1 regulates centriole duplication through controlling PLK4 levels

AUTHORS: Andrew Phillip Porter, Hannah Reed, Gavin R. M. White, Erinn-Lee Ogg, Helen J Whalley, and Angeliki Malliri
ARTICLE TYPE: Research Article

We have now reached a decision on the above manuscript.

To see the reviewers' reports and a copy of this decision letter, please go to: https://submit-jcs.biologists.org and click on the 'Manuscripts with Decisions' queue in the Author Area. (Corresponding author only has access to reviews.)

As you will see, the reviewers gave favourable reports but raised some critical points that will require amendments to your manuscript. I hope that you will be able to carry these out, because I would like to be able to accept your paper.

We are aware that you may be experiencing disruption to the normal running of your lab that makes experimental revisions challenging. If it would be helpful, we encourage you to contact us to discuss your revision in greater detail. Please send us a point-by-point response indicating where you are able to address concerns raised (either experimentally or by changes to the text) and where you will not be able to do so within the normal timeframe of a revision. We will then provide further guidance. Please also note that we are happy to extend revision timeframes as necessary.

Please ensure that you clearly highlight all changes made in the revised manuscript. Please avoid using 'Tracked changes' in Word files as these are lost in PDF conversion.

I should be grateful if you would also provide a point-by-point response detailing how you have dealt with the points raised by the reviewers in the 'Response to Reviewers' box. Please attend to all of the reviewers' comments. If you do not agree with any of their criticisms or suggestions please explain clearly why this is so.

Reviewer 1

Advance summary and potential significance to field

Porter and collaborators propose the involvement of Tiam1, a GEF of RAC1, on the regulation of Plk4 levels and subsequently in the centriole duplication.
I recognise the authors effort to address most of the reviewers’ comments, but I still have a last remark.
I think this work will be of general interest for the readers of the Journal of Cell Science. Therefore, I support the publication of this article.

Comments for the author

The data in supplementary figure 5, specifically figure 5b are not discussed in the manuscript. The percentage of centrosome amplification following three days of Tiam1 siRNA knockdown in U2OS

© 2021. Published by The Company of Biologists under the terms of the Creative Commons Attribution License (https://creativecommons.org/licenses/by/4.0/).

and HCT116 cells is shown in this table. One can see that cells with extra centrosomes do not exceed 3.8% in HCT116 and 6.0% in U2OS lines. These results differ from the data presented in the main figures. It would be essential to explain these results and understand the extent of the Tiam1 role in the centriole duplication the authors proposed.

Reviewer 3

Advance summary and potential significance to field

The authors addressed my main concerns. I now fully support the publication of this manuscript at Journal of Cell Science.

Comments for the author

.

Second revision

Author response to reviewers' comments

Response to Reviewers

We thank the reviewers for reconsidering our manuscript. We are pleased to read that we have addressed the comments raised in the first round of revision. With respect to the point now raised by Reviewer 1, we have addressed this as detailed below.

Reviewer 1 Comments for the Author:
The data in supplementary figure 5, specifically figure 5b are not discussed in the manuscript. The percentage of centrosome amplification following three days of Tiam1 siRNA knockdown in U2OS and HCT116 cells is shown in this table. One can see that cells with extra centrosomes do not exceed 3.8% in HCT116 and 6.0% in U2OS lines. These results differ from the data presented in the main figures. It would be essential to explain these results and understand the extent of the Tiam1 role in the centriole duplication the authors proposed.

We thank the Reviewer for pointing this out and prompting us to clarify this point. We have now altered the manuscript accordingly: we moved the data from Supplementary Figure 5a and 5b to Supplementary Figure 2, which allows us to better discuss this data within the context of the other data presented there. (We also make sure to provide a correct reference to both the representative images (new Supplementary Figure 2d) and quantitation (new Supplementary Figure 2e)). In describing the data, we state that while the level of centrosome amplification is small, it does indicate that some of the centrioles being produced are capable of producing fully functional centrosomes.

With respect to the amount of centrosome amplification being lower than that shown for centriole duplication, these are measuring separate (although related) phenotypes. The difference is addressed through comments in the discussion (which were included in the previous round of revisions). Now with reference to the centrosome amplification data being clearer in the main text, the discussion should be easier to follow.

© 2021. Published by The Company of Biologists under the terms of the Creative Commons Attribution License (https://creativecommons.org/licenses/by/4.0/).

Third decision letter

MS ID#: JOCES/2020/252502

MS TITLE: The RAC1 activator Tiam1 regulates centriole duplication through controlling PLK4 levels

AUTHORS: Andrew Phillip Porter, Hannah Reed, Gavin R. M. White, Erinn-Lee Ogg, Helen J Whalley, and Angeliki Malliri
ARTICLE TYPE: Research Article

I am happy to tell you that your manuscript has been accepted for publication in Journal of Cell Science, pending standard ethics checks.

© 2021. Published by The Company of Biologists under the terms of the Creative Commons Attribution License (https://creativecommons.org/licenses/by/4.0/).
